# Hypertonicity induces mitochondrial extracellular vesicles (MEVs) that activate TNF-α and β-catenin signaling to promote adipocyte dedifferentiation

**DOI:** 10.1186/s13287-023-03558-3

**Published:** 2023-12-20

**Authors:** Guopan Liu, Ying Wang, Yilin Pan, Li Tian, Ming Ho Choi, Li Wang, Jin Young Kim, Jian Zhang, Shuk Han Cheng, Liang Zhang

**Affiliations:** 1 Department of Biomedical Sciences, College of Veterinary Medicine and Life Sciences, City University of Hong Kong, 83 Tat Chee Avenue, Kowloon, Hong Kong China; 2 Key Laboratory of Biochip Technology, Biotech and Health Centre, Shenzhen Research Institute of City University of Hong Kong, Shenzhen, 518057 China; 3 Medicinal Chemistry and Bioinformatics Center, Shanghai Jiao Tong University, School of Medicine, Shanghai, 200025 China

**Keywords:** Multipotent stem cells, Adipocyte dedifferentiation, Hypertonic treatment, Mitochondrial EVs, TNF-α, Wnt/β-catenin signaling

## Abstract

**Background:**

Recent studies demonstrated that elevated osmolarity could induce adipocyte dedifferentiation, representing an appealing procedure to generate multipotent stem cells. Here we aim to elucidate the molecular mechanisms that underlie osmotic induction of adipocyte reprogramming.

**Methods:**

To induce dedifferentiation, the 3T3-L1 or SVF adipocytes were cultured under the hypertonic pressure in 2% PEG 300 medium. Adipocyte dedifferentiation was monitored by aspect ratio measurement, Oil Red staining and qPCR to examine the morphology, lipid droplets, and specific genes of adipocytes, respectively. The osteogenic and chondrogenic re-differentiation capacities of dedifferentiated adipocytes were also examined. To investigate the mechanisms of the osmotic stress-induced dedifferentiation, extracellular vesicles (EVs) were collected from the reprograming cells, followed by proteomic and functional analyses. In addition, qPCR, ELISA, and TNF-α neutralizing antibody (20 ng/ml) was applied to examine the activation and effects of the TNF-α signaling. Furthermore, we also analyzed the Wnt signaling by assessing the activation of β-catenin and applying BML-284, an agonist of β-catenin.

**Results:**

Hypertonic treatment induced dedifferentiation of both 3T3-L1 and the primary stromal vascular fraction (SVF) adipocytes, characterized by morphological and functional changes. Proteomic profiling revealed that hypertonicity induced extracellular vesicles (EVs) containing mitochondrial molecules including NDUFA9 and VDAC. Functionally, the mitochondrial EVs (MEVs) stimulated TNF-α signaling that activates Wnt-β-catenin signaling and adipocyte dedifferentiation. Neutralizing TNF-α inhibited hypertonic dedifferentiation of adipocytes. In addition, direct activation of Wnt-β-catenin signaling using BML-284 could efficiently induce adipocyte dedifferentiation while circumventing the apoptotic effect of the hypertonic treatment.

**Conclusions:**

Hypertonicity prompts the adipocytes to release MEVs, which in turn enhances the secretion of TNF-α as a pro-inflammatory cytokine during the stress response. Importantly, TNF-α is essential for the activation of the Wnt/β-catenin signaling that drives adipocyte dedifferentiation. A caveat of the hypertonic treatment is apoptosis, which could be circumvented by direct activation of the Wnt/β-catenin signaling using BML-284.

**Supplementary Information:**

The online version contains supplementary material available at 10.1186/s13287-023-03558-3.

## Background

With a mesenchymal origin, adipocytes are recognized as an essential energy storage entity as well as a critical endocrine source that regulates systemic metabolism [1]. Despite the essential physiological functions, hypertrophy and hyperproliferation of adipose tissues lead to obesity, which is a major risk factor for a series of diseases including dyslipidemia, cardiovascular disease, type 2 diabetes and cancer [2–4]. At the cellular level, obesity is underlined by the deregulation of adipogenesis, the differentiation of committed pre-adipocytes into mature adipocytes [5, 6]. Interestingly, adipocytes do not remain at a static stage of terminal differentiation after adipogenesis. Instead, mature adipocytes display a high degree of plasticity and can be reverted to a multipotent progenitor-like state [1], which is termed adipocyte dedifferentiation and has been broadly observed in vitro and in vivo [7]. For example, during late pregnancy and lactation, mammary adipocytes lose lipid droplets and dedifferentiate into fibroblast-like preadipocytes that later re-differentiate into adipocytes after lactation [8]. Moreover, recent studies observed adipocyte dedifferentiation in the development of different malignant tumors, which was associated with the activation of Wnt signaling [9, 10] and Notch signaling [11]. A better understanding of adipocyte differentiation/de-differentiation balance holds the key to effectively preventing/treating obesity and related diseases.

The mechanisms of adipogenesis have been extensively investigated [12, 13]. However, much less is known about the molecular mechanisms underlying adipocyte dedifferentiation. Generally, pro- and anti-adipogenic genes/pathways play opposite roles in adipocyte dedifferentiation. For example, C/EBPβ and PPARγ are key pro-adipogenic genes and are typically suppressed to allow adipocyte dedifferentiation. As a PPARγ agonist, rosiglitazone could block adipocyte dedifferentiation and tumor development in the Notch-driven liposarcoma mouse model [11]. In contrast, canonical Wnt/β-catenin signaling ligands are recognized as anti-adipogenic factors and have been identified as enhancers of dedifferentiation. While overexpression of Wnt1 gene inhibited adipogenic differentiation of progenitor cells, exogenous application of Wnt3a could induce dedifferentiation of both mouse 3T3-L1 and human adipocytes [9, 14, 15]. In addition to Wnt signaling, Notch, TGF-β and TNF-α signaling have all been reported to suppress adipogenesis and promote adipocyte dedifferentiation in various contexts [11, 15–17]. The detailed mechanism of how different signaling pathways cooperatively regulate adipocyte differentiation warrants further investigation.

Several methods have been established to induce adipocyte dedifferentiation, facilitating the investigation of molecular and cellular mechanisms. The “ceiling culture” of mature adipocytes was originally reported to induce the loss of lipid droplets and the emergence of progenitor-like cells, namely dedifferentiated fat cells (DFAT cells) [18]. Activation of TGF-β1 signaling and different collagens were shown to regulate DFAT cells of the ceiling culture system [19]. Recently, physical changes in microenvironment, including matrix stiffening, increased compressive force, and elevated osmotic pressure, have been shown to affect the cell fate and adipocyte dedifferentiation [20, 21]. Accordingly, hypertonic treatment has been shown to induce osmotic stress and promote adipocyte dedifferentiation [22]. Given the ample cellular abundance and the simple operation, hypertonicity-induced DFAT holds a strong promise to transform regenerative medicine [23]. Mechanistically, activation of the anti-adipogenic Wnt/β-catenin signaling was observed during the osmotic reprogramming of adipocytes. However, it remains elusive how hypertonicity activates the Wnt/β-catenin signaling and drives the dedifferentiation.

In this study, we investigated the mechanism underlying hypertonicity-induced reprogramming of adipocytes. We first established a robust cellular model whereby high-osmolarity could efficiently induce dedifferentiation of 3T3-L1 and stromal vascular fraction (SVF)-derived adipocytes. We then conducted a proteomic profiling of extracellular vesicles (EVs) released from the dedifferentiating adipocytes receiving hypertonic treatment. Intriguingly, this showed that hypertonicity prompts the adipocytes to eject mitochondria via EVs, which in turn enhance the secretion of TNF-α cytokine during the stress response. Importantly, hypertonicity-induced EVs and TNF-α are critical for the activation of the Wnt/β-catenin signaling that drives adipocyte dedifferentiation. Overall, our study defined a novel mitochondria-TNF-α axis that underlies the osmotic regulation of Wnt/β-catenin signaling and adipocyte dedifferentiation.

## Methods

### Cell culture

3T3-L1 (ATCC CL-173) cells were cultured in DMEM medium (Thermo Fisher, product no. 11965126) containing 10% FBS and 1% penicillin − streptomycin (all obtained from Thermo Fisher).

### Stromal vascular fraction (SVF) isolation

All animal experiments were performed in accordance with the protocol approved by the Institutional Animal Research Ethics Sub-Committee of City University of Hong Kong and Department of Health of the Hong Kong Special Administrative Region. C57BL/6 J mice were used for this study and maintained in a 12:12 h light–dark cycle. 6 to 8-week-old females were anesthetized with 4% of isoflurane by inhalation, and then inguinal, retroperitoneal WAT were removed. The tissues were washed in sterile PBS, shredded and incubated in collagenase type II (Sigma, product no. C6885) for 1 h at 37 °C before passed through a sterile 100 µm filter. SVF was obtained after 5 min centrifugation at 1000 rpm. Culture medium (DMEM supplemented with 10% FBS and 1% penicillin − streptomycin (all obtained from Thermo Fisher) was added to resuspend the cells, followed by culturing in 100 mm culture dishes. The following day, the culture medium was changed to remove the dead and non‐adherent cells.

### 3T3‐L1 and SVF differentiation

1.25 × 10^5^ of 3T3-L1 were seeded in 6-well dishes and cultured at 37 °C and 5% CO_2_. To begin the differentiation, 10 μg/ml insulin (MedChemExpress, product no. HY-P0035), 1 μM dexamethasone (MedChemExpress, product no. HY-14648), 0.5 mM 3-isobutyl-1-methylxanthine (IBMX) (MedChemExpress, product no. HY-12318) and 2 μM rosiglitazone (Sigma, product no. R2408-10MG) (Differentiation medium) were supplemented to the culture medium to constitute the differentiation medium for 2 days. For differentiation of SVF, the SVF cells were incubated for 5 days with differentiation medium. The medium was then changed to DMEM containing 10% FBS, 1% penicillin − streptomycin, and 10 μg/ml insulin for 48 h followed by changing to the culture medium.

### Oil Red O staining

Adipocytes were stained with Oil red O staining kit (Solarbio, product no. G1262) solution to detect the lipid droplets according to manufacturer’s instructions. Specifically, cells were fixed with 10% formalin for 20 min at room temperature, followed by washing with 60% isopropanol for 5 min. Subsequently, staining was conducted with ORO solution for 20 min at room temperature to enable binding of the dye to the lipid droplets. Finally, the cells were washed with distilled water to eliminate any residual dye.

### Hypertonic-induce dedifferentiation of adipocytes

To induce hypertonic shock, adipocytes were treated with a medium containing 2% polyethylene glycol 300 (PEG 300) diluted in culture medium. Specifically, adipocytes were exposed to the hypertonic dedifferentiation medium, composed of DMEM supplemented with 10% FBS and 1% penicillin–streptomycin (all obtained from Thermo Fisher) and 2% PEG-300 (MedChemExpress, product no. HY-Y0873), and maintained at 37 °C and 5% CO_2_. The medium was changed every 3 days, being careful to avoid shaking the dish to allow the hypertonic medium to diffuse slowly in the culture dish.

### Osteogenic differentiation

To induce osteogenic differentiation, 1 × 10^6^ cells were seeded in 6-well plates and incubated overnight at 37 °C and 5% CO_2_. The culture medium was then replaced with StemPro Osteogenesis Differentiation medium (Thermo Fisher, product no. A1007201) and cells were maintained in this medium for up to 14 days, with medium renewal every 3 days. This medium contains specific factors that promote differentiation toward the osteogenic lineage, allowing the cells to deposit mineralized extracellular matrix and express osteogenic markers.

### Alkaline phosphatase assay

Alkaline phosphatase (ALP) staining was conducted on Day 9 of the feeder cell-mediated reprogramming process, following the manufacturer's instructions for the Alkaline Phosphatase Detection Kit (Sigma, product no. SCR004). The staining was performed to detect the activity of ALP, a marker of pluripotency, and to assess the success of the reprogramming process.

### MitoTracker staining

To stain cells with MitoTracker (CST, product no. FM 9074), the 1 mM stock solution should be diluted directly into normal growth media to achieve a staining concentration ranging from 400 nM. The live cells were incubated for 15 min at 37 °C. Following incubation, live imaging is necessary to visualize the staining pattern within the cells.

### qPCR gene expression analysis

Total RNA was isolated using the RNA Extraction Kit (Takara, product no. 9767) in accordance with the manufacturer's recommended protocol. The first-strand cDNA was synthesized using the PrimeScript RT Reagent Kit (Takara, product no. RR047A) for RT-PCR. In brief, a 10 μl system containing 2 μl of 5 × PrimeScript RT Master Mix (Perfect Real Time) and RNase-Free Distilled Water and RNA solution, with a total RNA amount of 500 ng, was transferred to the ABI ProFlex PCR System (2 × 96-well) for reverse transcription. RT-PCR was then conducted using the Ex-Taq PCR kit (Takara, product no. RR820A) following the manufacturer's instructions. All RT-qPCR primer sequences were included in Additional file 2: Table S1.

### Western blotting

To obtain lysates, cells were washed twice with cold PBS and lysed with 1 mL of RIPA buffer (Beyotime, product no. P0013B) at 4 °C for 1 h with gentle rotation. The lysate was subsequently centrifuged at 12,000*g* for 20 min at 4 °C, and the resulting supernatant was quantified the protein concentration. Protein extracts were then separated by SDS-PAGE and transferred onto a NC membrane (Bio-Rad, product no. 1620112). The membranes were incubated overnight at 4 °C with antibodies against VDAC (CST, product no. 4866 s, 1:1000), NDUFA9 (Abcam, product no. ab14713, 1:1000), CD81(Santa Cruz, product no. sc-166029, 1:1000), Non-phospho (Active) β-Catenin (CST, product no. 8814, 1:2000), β-Catenin (CST, product no. 9562, 1:1000) and β-actin (Santa Cruz, product no. sc-47778, 1:1000). Following incubation with the appropriate secondary antibody (Abcam, product no. ab205719;Thermo Fisher, product no. 31460, 1:4000), the protein bands were visualized using an enhanced chemiluminescence (ECL) kit (Bio-Rad, product no. 170–5061) and captured using a Bio-red ChemiDoc.

### Chemical treatments

To inhibit EV secretion, adipocytes were treated with 10 μM GW4869 (Sigma-Aldrich, product no. D1692) in isotonic and hypertonic cultures. For control treatments, an equivalent volume of DMSO was added. After two days, the media were collected for EVs isolation and analysis.

To mitigate mitochondrial stress, adipocytes were treated with 1 mM pyruvic acid (Sigma-Aldrich, product no. 107360) in hypertonic cultures. For control treatment, an equivalent volume of DMSO was added. The treated cells were subjected to MTG staining, and the media were collected for EVs isolation and analysis.

### EVs isolation and protein digestion

The 3T3-L1 adipocytes were treated by isotonic and hypertonic medium. After 48 h culture, conditional mediums were collected. The EVs were isolated by a sequential ultracentrifugation includes removing supernatant by 300 g for 10 min, 2000 g for 15 min, 10000 g for 70 min and collecting the sediment by 120000 g for 2 h. The pellets were washed once with PBS and precipitated at 120000 g for 70 min. Purified EVs pellets were vacuum dried and then were resuspended in 100 μL of buffer containing 8 M urea (Sigma, product no. U1250) and 50 mM NH_4_HCO_3_ (Alfa Aesar, product no. 14249). The resuspended suspensions contain ~ 80 μg protein each as determined by DC protein assay (Bio-Rad, product no. 5000114). In-solution digestion was performed for the following LC–MS/MS analysis. Briefly, the EVs protein suspensions were reduced with 20 mM DTT for 10 min at 95 °C, followed by alkylation with 50 mM IAA for 30 min at RT in dark. The solutions were then diluted with 50 mM NH_4_HCO_3_ buffer (pH 8.0) to make the urea concentration lower than 1 M. MS-grade trypsin (Thermo Fisher, product no. 90058) coupled with Lyc-C protease was added into the solution (pH 8.0) at an enzyme/protein ratio of 1:25 to digest the protein at 37 °C overnight. The resulting peptide samples were desalted using C18 tips (Thermo Fisher, product no. 87784) and resuspended with 0.1% formic acid buffer (Thermo Fisher, product no. 85178) for LC–MS/MS analysis.

### LC–MS/MS analysis

The peptide samples were analyzed using EASY-nLC 1200 system combined with a Q Exactive HF mass spectrometry (Thermo Scientific). For each injection, 6 μL loading sample containing around 0.5 μg peptide was separated by a C18 nano-column (250 nm, 75 μm, 3 μm, PepSep, Denmark) (Thermo Fisher, product no. 87784) at a flow rate of 250 nl/min. The 75 min reversed-phase gradient was achieved by mobile phase A (0.1% formic acid in ultrapure water) and mobile phase B (0.1% formic acid/80% acetonitrile in ultrapure water). MS recording was operated in the range of 350 to 1800 m/z with a mass resolution of 120,000. The positive ion mode was employed with the spray voltage at 2000 V and a spray temperature of 320 °C. The resolution of dd-MS2 was 30,000 with a 1 × 10^5^ of AGC target. The Maximum IT was set at 60 ms, and the loop count was 12. The isolation window was 1.6 m/z, and the fixed first mass was 120.0 m/z.

### Database search

Raw files created by XCalibur 4.0.27 (Thermo Fisher) software were analyzed using Proteome Discoverer software (version 2.2, Thermo Fisher) against the UniProt mouse protein database in Sequest HT node. The precursor and fragment mass tolerances were set to 10 ppm and 0.02 Da, respectively. A maximum of two missed cleavage sites of trypsin was allowed. Carbamidomethylation (C) was set as static modification, and oxidation (M) and acetyl (protein N terminal) were set as variable modifications. The false-discovery rates (FDRs) of peptide spectrum matches (PSMs) and peptide identification were determined using the Percolator algorithm at 1% based on q value. For label-free quantification, the Minora Feature Detector node was used in the processing workflow, and the Precursor Ions Quantifier node and the Feature Mapper node in the consensus workflow. Normalization of the quantitative values was performed in Proteome Discoverer, based on the total peptide intensity of the samples.

### Transmission electron microscopy analysis of extracellular vesicle

EVs were fixed with a solution of 5% formaldehyde and 2% glutaraldehyde in 0.1 M cacodylate buffer (pH 7.2) overnight at 4 °C. After washing with 0.1 M cacodylate buffer, the samples were further fixed with a solution of 2% osmium tetroxide in 0.1 M cacodylate buffer for 2 h at room temperature, in the dark. Following another wash with 0.1 M cacodylate buffer, the samples were dehydrated using a series of ethanol solutions with increasing concentrations (30%, 50%, 70%, 80%, 90%, 95%, and 100%) and further dehydrated with 70% and 100% acetone. The samples were then infiltrated with Spurr's resin at increasing concentrations of 25%, 50%, 75%, and 100%. After polymerization for 2 days at 70 °C, ultrathin sections were prepared using a diamond knife, collected onto butvar-coated 100 mesh grids (EMS, product no. FF100-CU), and counterstained with UranyLess (EMS, product no. 22409) for 10 min and lead citrate for 1 min. Finally, the samples were imaged using a transmission electron microscope (Philips Technai 12) to visualize the internal structure and morphology of the EVs.

## Results

### Hypertonicity induced dedifferentiation of 3T3-L1 adipocytes

**Fig. 1:**
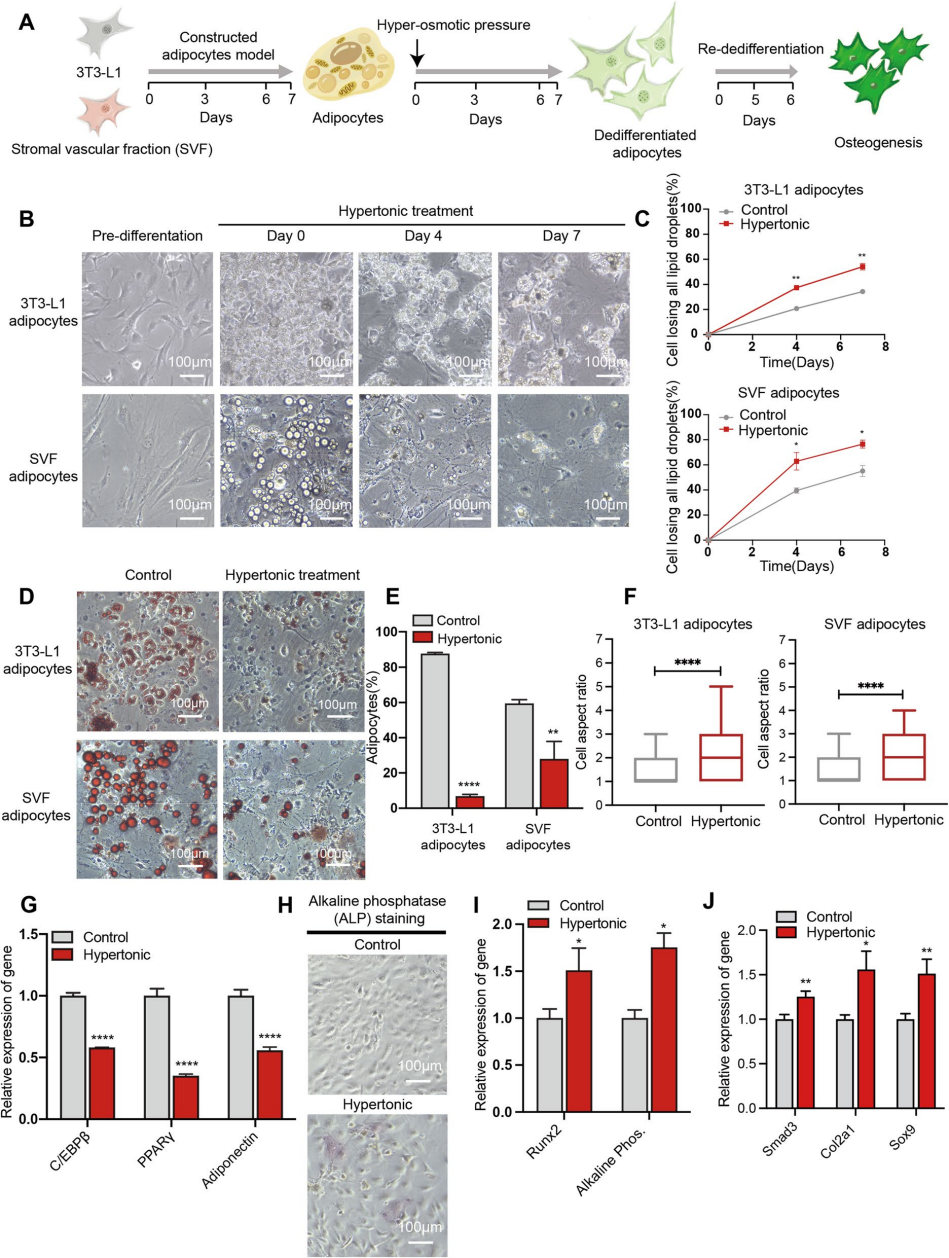
Hypertonic treatment induces dedifferentiation of adipocytes. **A** A diagram depicts the experimental workflow. Following the adipogenic culture of 3T3-L1 and SVF cells, the adipocytes were treated with the hypertonic medium for another seven days, and the dedifferentiated cells are isolated for osteogenic differentiation. **B** Representative images of adipocytes in the hypertonic culture for the indicated time. Scale bar: 100 µm. **C** Quantification of adipocytes losing clear lipid droplets over time in the indicated culture conditions. Error bars represent SD (*n* = 3 independent experiments). **D** and **E** Images (D) and quantification (E) of Oil Red O staining of adipocytes following indicated treatments for seven days. **F** Box blots depict the aspect ratio of indicated adipocytes (*n* = 200 cells) following control or hypertonic treatments of four days. **G** RT-qPCR analysis of the expression of C/EBPβ, PPARγ, and adiponectin in isotonic and hypertonic treated adipocytes. **H** Representative images of alkaline phosphatase (ALP) staining showing the osteogenic potential of the de-differentiated 3T3-L1 adipocytes. **I** RT-qPCR analysis of the expression of Runx2 and ALP following osteogenic induction of isotonic and hypertonic treatment of 3T3-L1 adipocytes. **J** RT-qPCR analysis of the expression of Smad3, Col2a1 and Sox9 following chondrogenic induction of isotonic and hypertonic treatment of 3T3-L1 adipocytes

Previous studies have reported that the physical compression induced by hypertonic treatment could lead to dedifferentiation of adipocytes derived from primary sources [21]. However, this phenomenon has not been recapitulated in cell line models. To address this, we examined whether hypertonic treatment could induce dedifferentiation of the well-established 3T3-L1 adipocytes model. We included adipocytes derived from the stromal vascular fraction (SVF) as the positive control. The experimental flow is illustrated in Fig. 1A. Briefly, 3T3-L1 and SVF cells were subject to adipogenic differentiation for 7 days, resulting in adipocytes with visible accumulation of lipid droplets and round morphology. We then started the dedifferentiation process of the adipocytes using hypertonic treatment with 2% PEG-300 in culturing media as describe before [21, 24]. Over four to seven days of hypertonic treatment, about 40–70% of the 3T3-L1 and SVF adipocytes lost lipid droplet, which were significantly higher than the control isotonic treatments (Fig. 1B, C). At the end of the 7-day treatment, we conducted Oil Red O staining which confirmed the depletion of lipid contents (Fig. 1D) and adipocytes (Fig. 1E). In parallel, we observed a significant increase in the aspect ratios of cells in the hypertonic treatment group (Fig. 1F), which is in line with the elongated morphologies of dedifferentiated adipocytes reported in previous studies [9, 25]. To validate the dedifferentiation of the 3T3-L1 adipocytes, we examined the expression of adipocyte marker genes. Compared with the isotonic control, hypertonic treatment of 3T3-L1 adipocytes led to 40–60% reductions in the expression of adipogenic markers, including C/EBPβ, PPAR-γ, and adiponectin (Fig. 1G). In parallel, we applied immunostaining to examine CD13 and Endoglin, which are surface markers of dedifferentiated adipocytes. Quantitative analysis revealed significantly higher expression of CD13 and Endoglin in adipocytes of the hypertonic treatment than the isotonic control (Additional file 2: Fig. S1A–D). Overall, these results indicated that hypertonic treatment could effectively induce dedifferentiation of 3T3-L1 adipocytes.

An important functional feature of the dedifferentiated adipocytes is the ability to re-differentiate into other mesenchymal lineages. Next, we evaluated the 3T3-L1 adipocytes for their capacity in osteogenic and chondrogenic re-differentiation after hypertonic treatment. Osteogenic induction promoted the expression of alkaline phosphatase (ALP), a marker of the osteogenic lineage, in 3T3-L1 adipocytes receiving hypertonic treatment (Fig. 1H–I). This was accompanied by significantly increased expressions of Runx2, which is another osteogenic marker (Fig. 1I). In addition, chondrogenic induction of the 3T3-L1 adipocytes after hypertonic treatment led to an increased expression of chondrogenic marker genes, including Smad3, Col2a1 and Sox9 (Fig. 1J). Importantly, the control cells that received isotonic treatment did not display osteogenic nor chondrogenic differentiation capacity, in line with the adipogenic commitment of the 3T3-L1 cells. Taken together, we conclude that hypertonic treatment could effectively induce the multipotent dedifferentiation of 3T3-L1 adipocytes.

### Adipocytes receiving hypertonic treatment eject mitochondria in extracellular vesicles

**Fig. 2:**
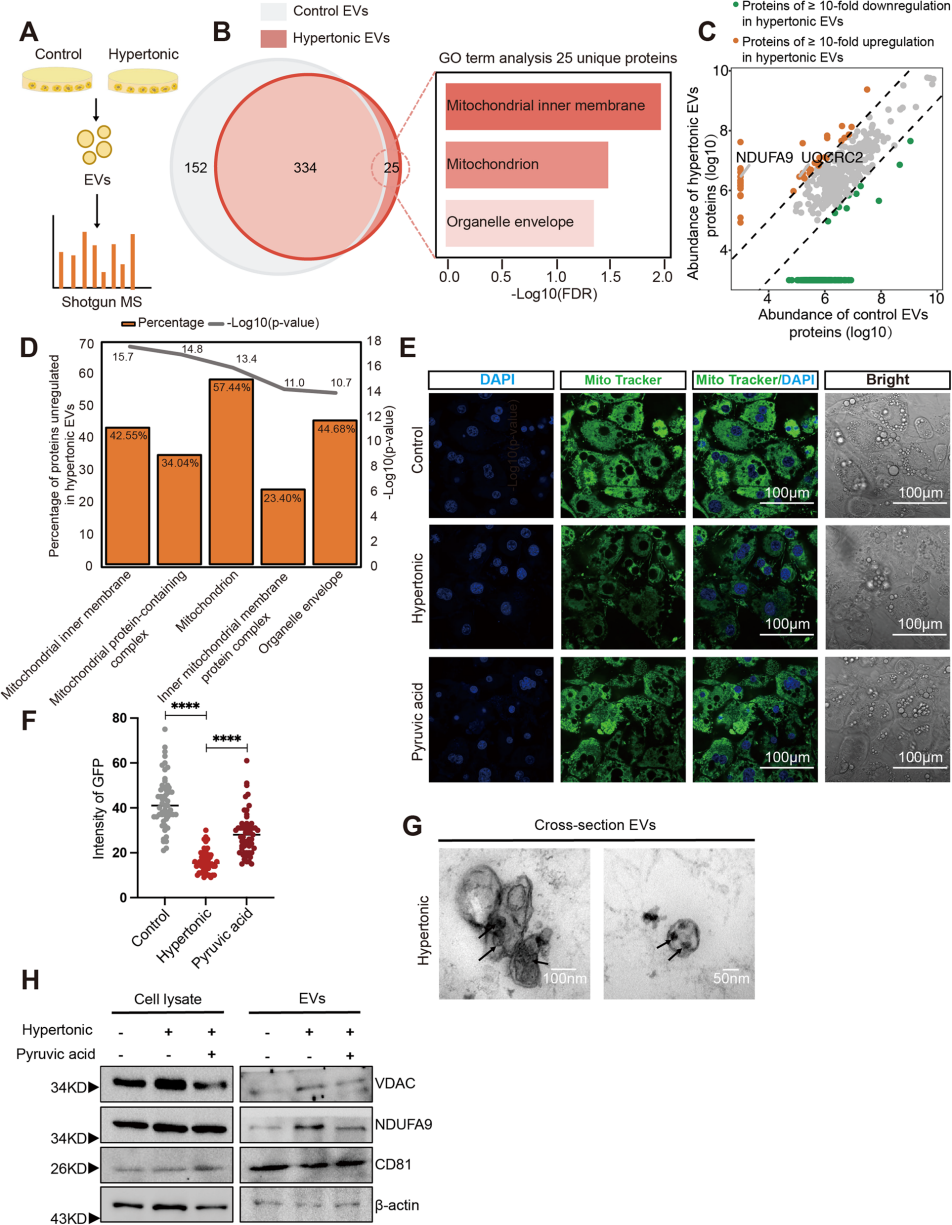
Hypertonic treatment prompts adipocytes to eject mitochondria via EVs. **A** EVs from isotonic control and hypertonic culture of 3T3L1 adipocytes were subject to quantitative proteomic analysis. **B** Venn diagram compares the proteins of control and hypertonic EVs from adipocytes. The right graph depicts the GO-term analysis of the 25 hypertonic-unique proteins. **C** Scatter plot summarizes the differential analysis of the EVs proteome between the control and hypertonic pressure. Sloping dotted lines demarcate tenfold difference in the abundance. **D** GO-term analysis of the up-regulated proteins in the hypertonic EVs. The top five cellular components are shown. **E** and **F** The 3T3-L1 adipocytes receiving indicated treatments for 8 h were stained using MitoTracker Green (green) and DAPI (blue). Relative intensities of the green channel were quantified. Scale bars, 10 µm. **G** Representative transmission electron microscopy (TEM) images of hypertonic EVs from 3T3-L1 adipocytes. Arrows indicate inclusions of mitochondria fragments. **H** Cell lysates and EVs of 3T3-L1 adipocytes following indicated treatments were subject to immunoblot analyses of the indicated proteins, respectively. Full-length blots are presented in Additional file 1: Additional file 1

Cellular transformation is commonly accompanied by autocrine signaling activities [26]. Among them, extracellular vesicles (EVs) are a prominent mediator of cell–cell communication in many biological contexts including cancer [27]. To investigate whether EV factors may regulate the hypertonic dedifferentiation of adipocytes, we isolated the EVs from the isotonic or hypertonic treatments of 3T3-L1 adipocytes and compared their proteomic contents using LC–MS analysis (Fig. 2A). Overall, we identified 359 and 486 proteins (identified with ≥ 2 unique peptides) in the EVs from hypertonic- and isotonic-treated 3T3-L1 adipocytes, respectively. While 334 proteins were shared between the two EVs population, we observed 25 proteins that are uniquely present in the hypertonic EVs (Fig. 2B). Intriguingly, Gene Ontology (GO) term annotation of the 25 hypertonic-unique proteins revealed enrichment of mitochondrial components (Fig. 2B; Additional file 2: Table S2), which host the enzymatic complexes of oxidative phosphorylation and play a key role in ATP production and energy homeostasis of cells. In addition, quantitative analysis identified 47 proteins with > tenfold upregulation in hypertonic EVs (Fig. 2C; Additional file 2: Table S3). Notably, GO term analysis ascribed more than 50% of the hypertonically up-regulated EV proteins as components of the mitochondrion (Fig. 2D), including NDUFA9 and UQCRC2, which are components of the NADH-ubiquinone oxidoreductase (complex I) and the ubiquinol-cytochrome c reductase (complex III) on the mitochondrial inner membrane, respectively. These results suggest that adipocytes may eject mitochondrial components through EVs in response to hypertonic treatment.

Hypertonicity is known to disrupt the ionic homeostasis of cells and reduce the electronic potential in mitochondria, consequentially inhibiting ATP production [28]. Therefore, we examined the status of mitochondria in adipocytes using MitoTracker Green (MTG), a fluorescent probe that accumulates in live-cell mitochondria in an electronic-potential-dependent manner [29]. Compared to the isotonic control, hypertonic treatment markedly blocked MTG staining of mitochondria in adipocytes (Fig. 2E, F). Pyruvic acid is known to alleviate mitochondrial stress by improving electronic potential [30, 31], and application of pyruvic acid (1 mM) to the hypertonic treatment effectively restored MTG staining (Fig. 2E, F). These results suggested that hypertonicity treatment could lead to disrupted electronic potential and mitochondrial stress in adipocytes.

Recent investigations have revealed that a spectrum of cells could eject damaged mitochondria from their cytosol [32, 33]. Therefore, we hypothesized that adipocytes eject mitochondria in EVs in response to the hypertonic stress of mitochondria. To address this, we exploited electronic microscopy (EM) to examine the EVs released from 3T3-L1 adipocytes under hypertonic treatment and observed folded membranous structures that resemble mitochondrial cristae enclosed in EVs (Fig. 2G). In parallel, hypertonic treatment also increased the levels of mitochondrial proteins VDAC and NDUFA9 in the EVs of 3T3-L1 adipocytes, which were reduced by pyruvic acid that alleviates mitochondrial stress (Fig. 2H). At the same time, we did not observe differences in the levels of the pan-EV marker CD81, indicating the specific effects on the mitochondrial components. In addition, GW4869, a compound that blocks EVs secretion, substantially inhibited the EVs level of NDUFA9 induced by hypertonic treatments (Additional file 2: Fig. S2). This supports that mitochondrial components were released through the EVs route during hyperosmotic stress. Taken together, these results suggest that hypertonic treatment leads to mitochondrial stress in adipocytes, resulting in ejection of mitochondrial components in EVs. We term these vesicles mitochondrial EVs (MEVs).

### MEVs activate TNF-α signaling in promoting the hypertonicity-induced adipocytes dedifferentiation

**Fig. 3:**
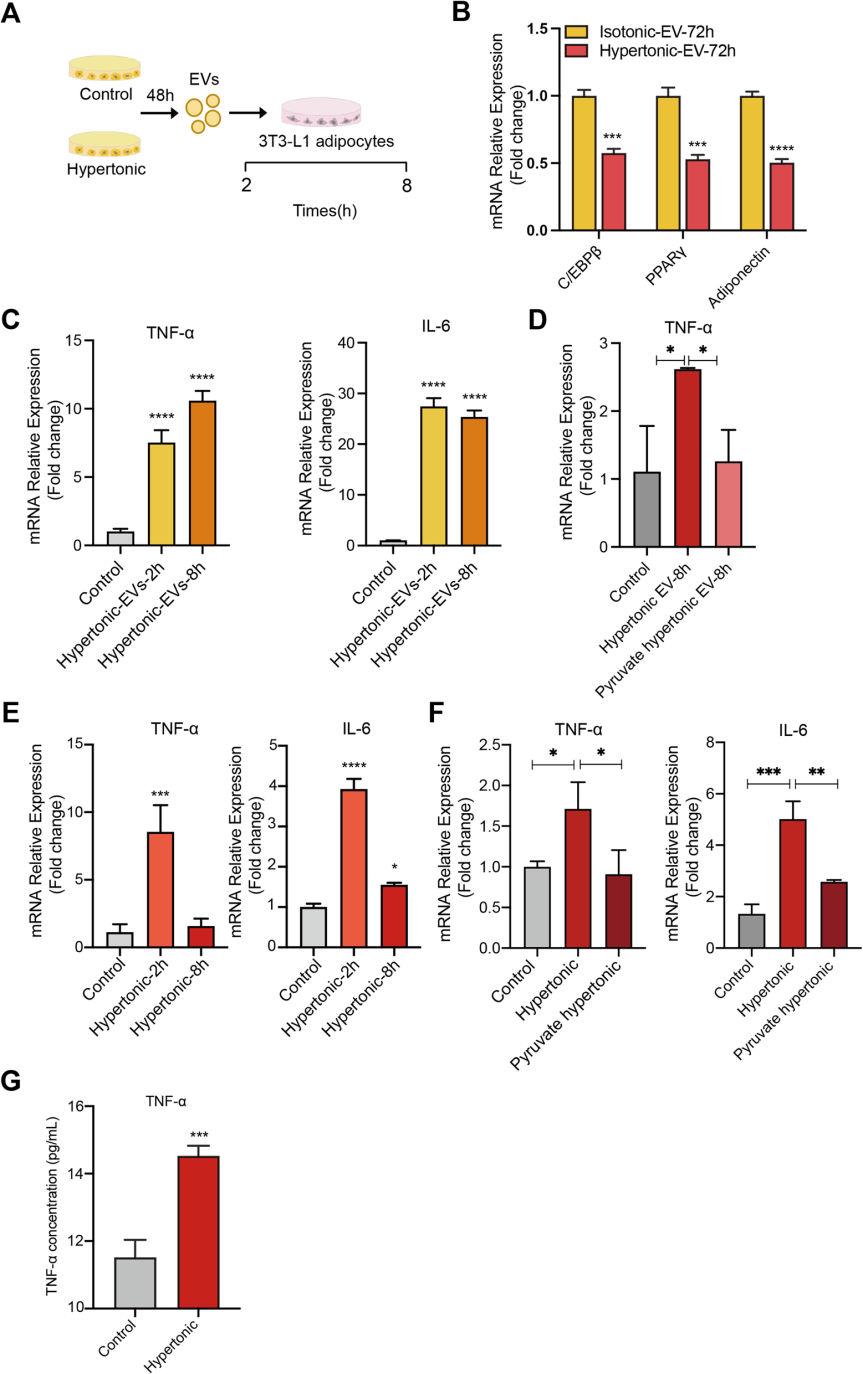
Hypertonicity and mitochondrial EVs (MEVs) activates TNF-α signaling in 3T3-L1 adipocytes. **A** EVs from control or hypertonic cultures of 3T3-L1 adipocytes were used to treat naïve 3T3-L1 adipocytes in isotonic culture. **B** RT-qPCR analysis of the expression of C/EBPβ, PPARγ, and adiponectin in 3T3-L1 adipocytes treated with EVs isolated from the indicated cultures. **C** RT-qPCR analysis of the expression of TNF-α and IL-6 in 3T3-L1 adipocytes treated with EVs isolated from the adipocytes from the indicated cultures. **D** RT-qPCR analysis of the expression of TNF-α in 3T3-L1 adipocytes receiving EVs from the indicated treatments. **E** RT-qPCR analysis of the expression of TNF-α and IL-6 in 3T3-L1 adipocytes receiving indicated treatments. **F** qPCR analysis of TNF-α related inflammatory factor in 3T3-L1 adipocytes receiving indicated treatments for 2 h.** G** ELISA analysis of the protein levels of TNF-α in the media of 3T3-L1 adipocytes receiving indicated treatments for 8 h

EV-mediated transfer of mitochondrial contents has been shown to stimulate metabolic and inflammatory responses of recipient cells [34]. Therefore, MEVs could constitute an autocrine factor that regulates adipocyte dedifferentiation. To test this, we isolated MEVs from the hypertonic treatment of 3T3-L1 adipocytes and applied them to naïve 3T3-L1 adipocytes cultured in isotonic condition (Fig. 3A). After 72 h, RT-qPCR analysis revealed a significant reduction of the expression of adipogenic markers, including C/EBPβ, PPAR-γ, and adiponectin (Fig. 3B). These results suggested a potential role of MEVs as the mediator of hypertonic-induced adipocyte dedifferentiation.

Extracellular mitochondria have been shown to promote inflammatory processes [35–37]. We next examined whether the hypertonic MEVs could influence the inflammatory pathways in adipocytes. Indeed, application of hypertonic MEVs to 3T3-L1 adipocytes significantly induced the expression of a series of inflammatory genes, including TNF-α, IL-6, RIP1, CEBPA, and MCP-1 (Fig. 3C; Additional file 2: Fig. S3A). In particular, a robust 4- and eightfold induction was observed for the key inflammatory genes TNF-α and IL-6 after 2-h MEVs treatment, respectively. The induction of both genes remained high at 8 h post treatment, suggesting the long-lasting effect of hypertonic MEVs. Importantly, reducing mitochondria stress by pyruvic acid prevented the hypertonic EVs from inducing TNF-α expression (Fig. 3D). Given that pyruvic acid decreases the mitochondrial contents in EVs, these results suggested that hypertonic MEVs could stimulate inflammatory signaling in adipocytes.

Next, we examined the inflammatory responses of adipocytes in the hypertonic culture. Similar to the hypertonic EVs treatment, 2 h of hypertonic culture induced the expression of inflammatory genes TNF-α, IL-6, RIP1, CEBPA, and MCP-1 in 3T3-L1 adipocytes (Fig. 3E; Additional file 2: Fig. S3B). Interestingly, the expression levels of TNF-α, IL-6, CEBPA, and MCP-1 declined substantially after 8 h of hypertonic culture. This contrasted with the long-lasting effect of hypertonic EVs and could be due to adaptation of adipocytes to the relatively mild hypertonicity. Notably, addition of pyruvic acid to the hypertonic culture abolished the induction of TNF-α and IL-6 expression (Fig. 3F), further supporting the role of mitochondrial stress in the effects of hypertonicity. Importantly, ELISA assay revealed an increased level of TNF-α protein in the media of hypertonic-treated adipocytes (Fig. 3G). Altogether, these results indicated that hypertonic stress and mitochondrial EVs could activate inflammatory signaling in adipocytes.

### TNF-α activates β-catenin to promote the hypertonicity-induced dedifferentiation of adipocytes

**Fig. 4:**
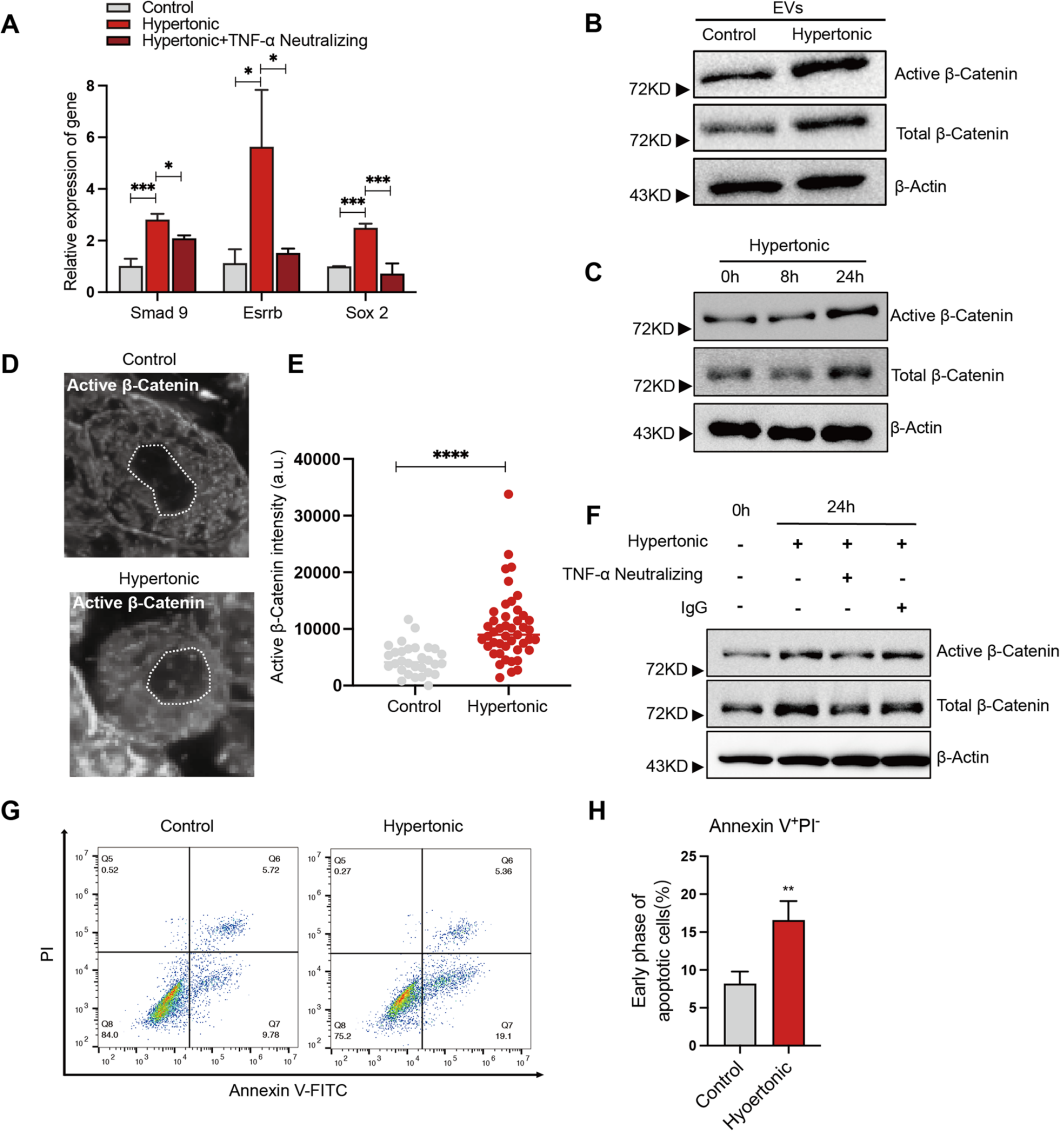
TNF-α activates β-catenin and apoptosis in adipocytes under hypertonic treatment. **A** RT-qPCR analysis of the expression of Smad9, Esrrb and Sox2 in adipocytes receiving the indicated treatments. **B** Total cell lysates from 3T3-L1 adipocytes treated by EVs from isotonic or hypertonic adipocyte cultures were subject to western blot analyses of indicated proteins. Full-length blots are presented in Additional file 1: Additional file 2. **C** Total cell lysates from 3T3-L1 adipocytes in indicated culture conditions were subject to western blot analyses of indicated proteins. Full-length blots are presented in Additional file 1: Additional file 3. **D** Representative images of immunostaining of active β-catenin in 3T3-L1 adipocytes following control or hypertonic treatment. **E** Quantification of the intensity of active β-catenin staining in the nucleus of adipocytes following control or hypertonic treatment for 24 h. **F** 3T3-L1 adipocytes were treated with the indicated conditions for 24 h and the cell lysates were subject to western blot analysis of indicated proteins. Full-length blots are presented in Additional file 1: Additional file 4. **G** Representative results of annexin V/PI flow cytometry analysis of adipocytes receiving indicated treatments. **H** Bar graphs depict the proportions of cells in early-phase apoptosis in adipocytes receiving indicated treatment

Previous studies have highlighted the anti-adipogenic activity of the TNF-α inflammatory signaling [17, 38]. To examine whether the role of TNF-α in hypertonicity-induced adipocyte dedifferentiation, we applied TNF-α neutralizing antibody to 3T3-L1 adipocytes in the hypertonic culture. Compared to the control antibody, TNF-α neutralizing antibody significantly inhibited the hypertonicity-induced expression of stem cell genes, including Esrrb, and Sox2 (Fig. 4A). Though not significant, a modest reduction of Smad9 was also observed after neutralizing TNF-α in the dedifferentiating adipocytes (Fig. 4A). These results pointed to a key role of TNF-α in hypertonicity-induced adipocyte dedifferentiation.

The Wnt/β-catenin pathway is also known to inhibit adipogenesis [39] and promote the dedifferentiation of mature adipocytes [9]. In fact, activation of β-catenin was reported to underlie hypertonic dedifferentiation of adipocytes [21]. We then assessed the hypertonic EVs treatment for the influence on β-catenin in adipocytes. Western blot demonstrated stabilization of β-catenin and accumulation of non-phosphorylated (active) β-catenin in 3T3-L1 adipocytes after 24 h of hypertonic MEVs treatment (Fig. 4B). This was also observed in 3T3-L1 adipocytes after 24 h of hypertonic culture (Fig. 4C). In parallel, immunostaining revealed that the nuclear accumulation of active β-catenin in hypertonic culture (Fig. 4D, E). Therefore, in addition to inducing TNF-α, hypertonic stress and the mitochondrial EVs also activate the anti-adipogenic Wnt/β-catenin signaling.

TNF-α has been reported to induce the stabilization of β-catenin and inhibit adipogenesis [17]. Therefore, we assessed the importance of TNF-α in hypertonic activation of β-catenin during dedifferentiation of adipocytes. Indeed, application of the TNF-α neutralizing antibody markedly block the stabilization of total β-catenin and non-phosphorylated (active) β-catenin in hypertonic culture (Fig. 4F). These results demonstrate that TNF-α is essential for the hypertonic activation of Wnt/β-catenin signaling that drives adipocyte dedifferentiation.

### Hypertonic treatment induces apoptosis of adipocytes that is circumvented by direct activation of β-catenin signaling

The hypertonic dedifferentiation of adipocytes typically requires up to 7 days of culture in 2% PEG-300. Given that TNF-α is also known as a proinflammatory cytokine to induce apoptosis of adipocytes [40], we decided to examine the apoptotic status of adipocytes using the AnnexinV-FITC/PI staining. Compared to the isotonic control, we observed a significantly increase of cells in the early phase of apoptosis indicated by the AnnexinV staining (Fig. 4G, H). At the same time, we did not observe a difference in PI-positive cells in the early phase of apoptosis, which could potentially be due to the loss of such cells in the washing process prior to the harvest. We concluded that the extended hypertonic treatment could lead to apoptosis in parallel to dedifferentiation of adipocytes.

**Fig. 5:**
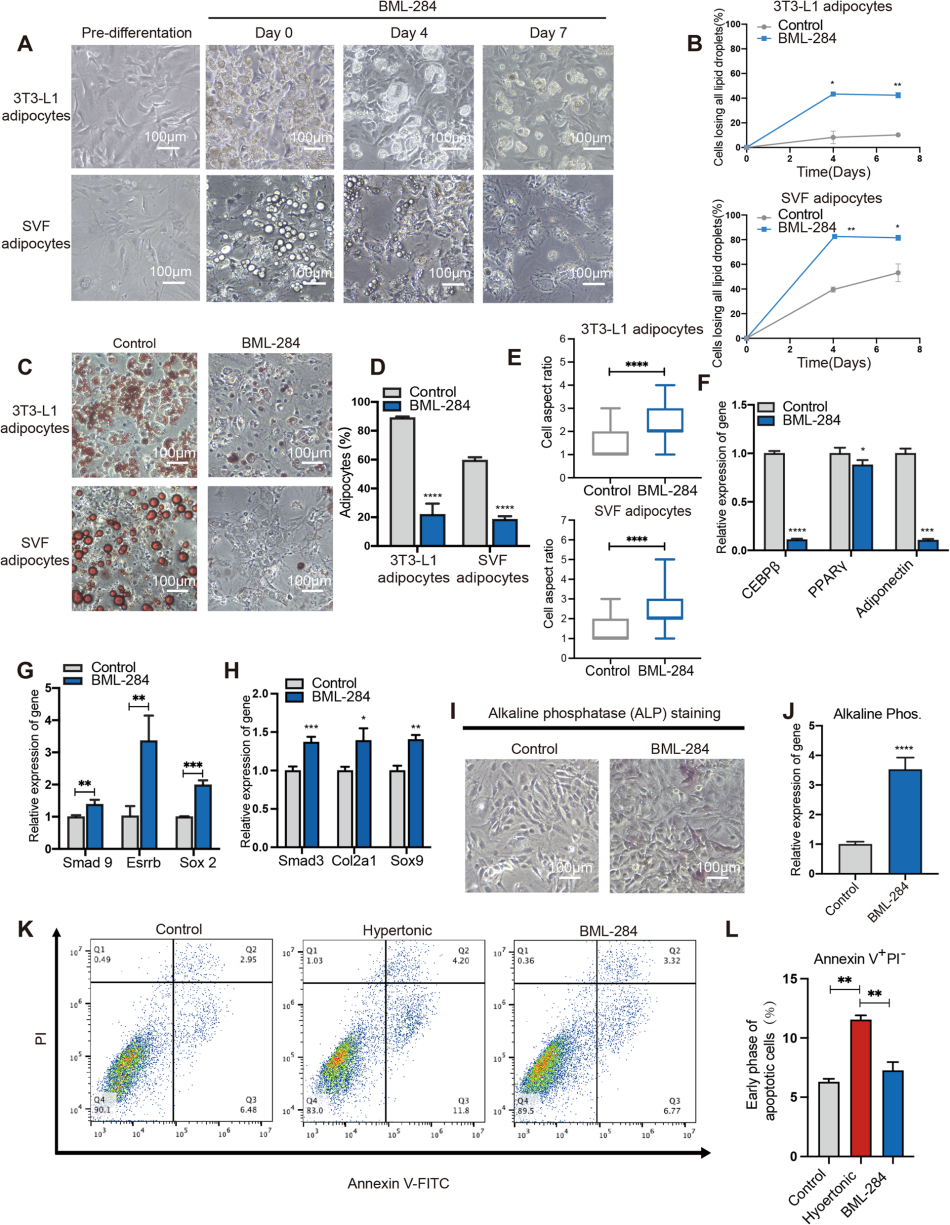
BML-284 induces dedifferentiation of adipocytes. **A** Representative images of adipocytes treated by BML-284 for the indicated time. Scale bar, 100 µm. **B** Quantification of adipocytes losing all their clear lipid droplets during seven days of control or BML-284 culture. Error bars represent SD (*n* = 3, independent repeats). **C** and **D** Images (C) and quantification (D) of Oil Red O show adipocytes losing all their clear lipid droplets following indicated treatments for seven days. **E** Box plots depict the aspect ratio of indicated adipocytes (*n* = 200 cells) following control or BML-284 treatments. **F** RT-qPCR analysis of the expression of C/EBPβ, PPARγ, and adiponectin in control or BML-284 treated adipocytes. **G** RT-qPCR analysis of the expression of pluripotency markers Smad9, Esrrb and Sox2 in control or BML-284 treated adipocytes. **H** RT-qPCR analysis of the expression of Smad3, Col2a1 and Sox9 of isotonic and BML-284 treated adipocytes following chondrogenic induction. **I** Representative images of alkaline phosphatase (ALP) staining showing the osteogenic potential of the de-differentiated 3T3-L1 adipocytes following control or BML-284 treatments. **J** RT-qPCR analysis of the expression of ALP following osteogenic induction of control- or BML-284-treated 3T3-L1 adipocytes. **K** Representative results of annexin V/PI flow cytometry analysis of adipocytes receiving indicated treatments. **L** Bar graphs depict the proportions of cells in early-phase apoptosis in adipocytes receiving indicated treatments

To circumvent the apoptotic effects of hypertonicity and TNF-α in inducing adipocyte dedifferentiation, we exploited BML-284, a small compound that stabilizes β-catenin and directly activates the Wnt/β-catenin signaling. We treated 3T3-L1 and SVF adipocytes in isotonic cultures with BML-284 (10 mM) and examined the lipid droplets and morphological changes. Strikingly, 4 days of BML-284 treatment led to a significant disappearance of visible lipid droplets (Fig. 5A, B). This is accompanied by significant increases of cells with elongated morphology in BML-284-treated 3T3-L1 and SVF adipocytes (Fig. 5C). These results indicated the anti-adipogenic activity of BML-284, which was further supported by decreased Oil red staining (Fig. 5D, E). In parallel, we observed significant decreases in the expression of adipogenic genes (C/EBPβ, PPAR-γ, and adiponectin) in conjunction to significant increases in the expression of marker genes (Smad9, Esrrb and Sox2) and proteins (CD13 and endoglin) of multipotency in BML-284 treated cells (Fig. 5F–G; Additional file 2: Fig. S4). Importantly, the re-differentiation assays revealed increased expression of osteogenic and chondrogenic markers in BML-284-treated adipocytes after inducing treatments correspondingly (Fig. 5H–J). Altogether, these results indicated that direct activation of β-catenin signaling by BML-284 could induce adipocyte dedifferentiation.

Next, we compared BML-284 and the hypertonic treatment for their effects on cell apoptosis. Notably, while the hypertonic treatment induced a significantly level of apoptosis in 3T3-L1 adipocytes, BML-284 did not lead to significant apoptosis (Fig. 5K, L). Overall, these results demonstrated that BML-284 treatment could effectively induce adipocyte dedifferentiation while circumventing the apoptotic effect of hypertonic treatment.

## Discussion

Multipotent dedifferentiation of adipocytes holds strong promises to revolutionize regenerative medicine. However, the application potential is hampered by the unclear mechanisms and low efficiency [41]. Our work defined a novel signaling axis that promotes the multipotent dedifferentiation of adipocytes. High osmolarity induce the mitochondrial stress and prompts the adipocytes to release of mitochondria-containing EVs (Fig. 2), which in turn enhances the secretion of TNF-α as a pro-inflammatory cytokine during the stress response (Fig. 3). Importantly, we revealed that the Wnt/β-catenin signaling was activated by TNF-α to drive the adipocyte dedifferentiation (Fig. 4). Furthermore, direct activation of Wnt/β-catenin signaling by BML-284 could efficiently induce adipocyte dedifferentiation while circumventing the apoptotic effect of the hypertonic treatment (Fig. 5). Our results provide a mechanistic foundation for efficiently inducing adipocyte differentiation as a strategy in regenerative medicine.

Osmotic stress can take place in a diverse array of conditions with physiologic or pathologic influences on the adipocytes [42]. For example, hyperosmotic stress of adipocytes was reported to inhibit insulin signaling and induce insulin-resistance [43]. In addition, Zhu et al. reported that the adipocytes in breast cancer tissues could undergo the adipocyte mesenchymal transition (AMT) that generate multiple cell types constituting an inflammatory and tumor-promoting stroma [44]. Although the mechanism of AMT remains unclear, it is important to note that the osmotic stress and compression in breast cancer have been reported to activate β-catenin signaling and induce the adipocytes dedifferentiation with multi-lineage redifferentiation potentials [21]. Our results suggest that hypertonic MEVs mediate β-catenin activation and adipocytes dedifferentiation in response to osmotic stress (Fig. 4). It would be of interest to investigate whether hypertonic stress of mitochondria and MEVs of adipocytes are implicated in the malignant AMT of breast adipose tissue in vivo. This could provide a potential therapeutic target in the breast cancer microenvironment.

**Fig. 6:**
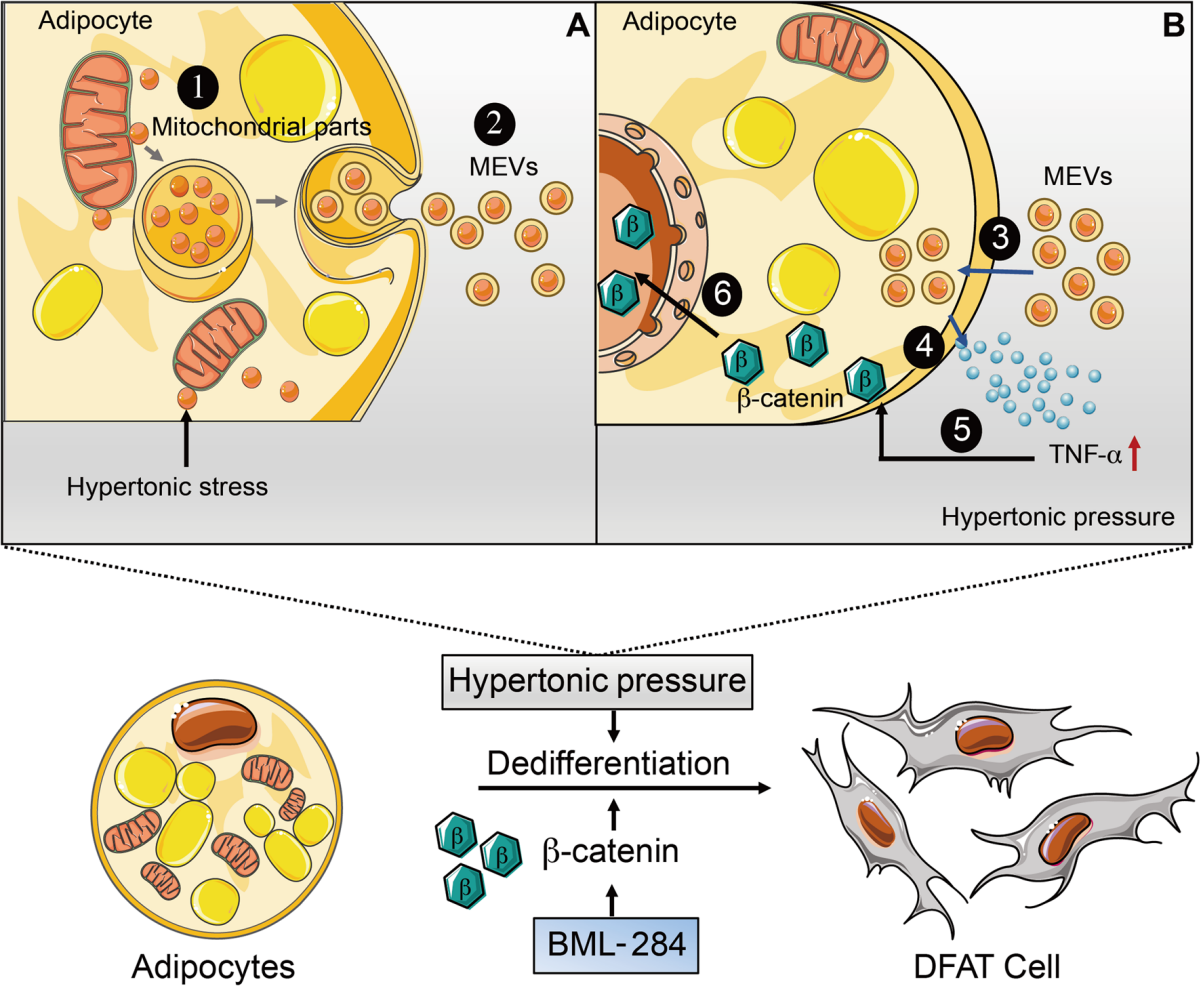
Working model of adipocyte dedifferentiation induced by hypertonicitys

Recent studies have demonstrated that various cells could eject mitochondria to regulate homeostasis. For example, Nicolás-Ávila et al. found that cardiomyocytes release damaged mitochondria in extracellular vesicles (EVs) to maintain proper heart function [45]. We here report that hypertonic treatment of adipocytes could lead to EV-mediated mitochondrial ejection. Mechanistically, the fluctuation of the cellular osmolarity could perturb the ion homeostasis and electronic potential in mitochondria, leading to damages of the components. This is supported by the data that pyruvate could reduce the hypertonic MEVs (Fig. 2). Similarly, Rosina et al. have reported that thermogenically stressed brown adipocytes eject dysfunctional mitochondrial parts in EVs to prevent failure of the thermogenic program [32]. It remains unclear about the cellular process and mechanisms of releasing mitochondrial components via EVs. It would be interesting to compare MEVs from different conditions for their components and activities in inducing adipocyte dedifferentiation. Accumulating evidence also indicate that adipocyte dedifferentiation plays an important role in tissue homeostasis. For example, Zhang et al. reported that dermal adipose tissue undergoes dedifferentiation and redifferentiation in hair cycle and wound healing [46]. In line with this, lineage tracing and single-cell RNA sequencing have revealed myofibroblasts derived from adipocytes during wound healing [47]. It is of interest to note that wound healing is accompanied by activation of the inflammatory cytokines including TNF-α and we found that the hypertonic MEVs promote the TNF-α → β-catenin signaling axis in driving adipocytes dedifferentiation. In the studies of both Nicolás-Ávila et al. and Rosina et al., immune cells such as macrophages play a critical role in removing the ejected mitochondria and maintaining the homeostasis of the source tissue. Therefore, how MEVs function with the inflammation/immune system to induce adipocyte dedifferentiation in vivo would need further investigation. In addition, we showed that hypertonicity also induces apoptosis in adipocytes, which could be alleviated by direct activation of β-catenin. This suggests heterogeneity in the responses of adipocytes toward hypertonicity and ejected MEVs. Elucidating the mechanisms that dictate the apoptosis/dedifferentiation fate would pave the way for effectively harnessing adipocytes for regenerative medicine (Fig. 6).

## Conclusion

Our study has shed new light on the mechanisms underlying the multipotent dedifferentiation of adipocytes, which holds great promise for regenerative medicine. We have identified a novel signaling axis involving high osmolarity-induced mitochondrial stress, EV-mediated mitochondrial ejection, TNF-α secretion, and Wnt/β-catenin activation that promotes adipocyte dedifferentiation. We also demonstrated that direct activation of Wnt/β-catenin signaling by BML-284 could efficiently induce adipocyte differentiation while circumventing the apoptotic effect of hypertonic treatment, providing a potential strategy for regenerative medicine applications.

### Supplementary Information

**Additional file 1.** Original western blot images.

**Additional file 2.** Supplementary Figures and Tables.

## Data Availability

The mass spectrometry proteomics data have been deposited to the ProteomeXchange Consortium via the iProX partner repository with the dataset identifier PXD04259. The data can be accessed through the permanent link: https://www.iprox.cn/page/project.html?id=IPX0005941000.

## Abbreviations

SVF: Stromal vascular fraction
EVs: Extracellular vesicles
MEVs: Mitochondrial extracellular vesicles
DFAT: Dedifferentiated fat cells
ACK: Ammonium–chloride–potassium
PEG 300: Polyethylene glycol 300
ALP: Alkaline phosphatase
C/EBPβ: CCAAT/enhancer binding protein beta
PPARγ: Peroxisome proliferator-activated receptor gamma
CD13: Aminopeptidase N
Runx2: RUNX Family Transcription Factor 2
Smad3: Mothers against decapentaplegic homolog 3
Col2a1: Collagen Type II Alpha 1 Chain
Sox9: SRY-Box Transcription Factor 9
ATP: Adenosine triphosphate
NDUFA9: NADH:Ubiquinone Oxidoreductase Subunit A9
UQCRC2: Ubiquinol-Cytochrome C Reductase Core Protein 2
VDAC: Voltage-dependent anion channel
CD81: Cluster of Differentiation 81
TNF-α: Tumor Necrosis Factor-α
IL-6: Interleukin 6
RIP1: Receptor-interacting serine/threonine-protein 1
CEBPA: CCAAT/enhancer-binding protein alpha
MCP-1: Monocyte chemoattractant protein-1
Esrrb: Estrogen Related Receptor Beta
Sox2: Sex determining region Y-box 2
Smad9: Mothers against decapentaplegic homolog 9
