# Supplementary material for: Hypertonicity induces mitochondrial extracellular vesicles (MEVs) that activate TNF-α and β-catenin signaling to promote adipocyte dedifferentiation
Source: Stem Cell Res Ther. 2023 Dec 20;14:333. doi: 10.1186/s13287-023-03558-3 (PMC10731851; doi:10.1186/s13287-023-03558-3)
Supplement: Supplementary file 1 — Additional file 1. Original western blot images. [file 13287_2023_3558_MOESM1_ESM.pdf]

Figure 2

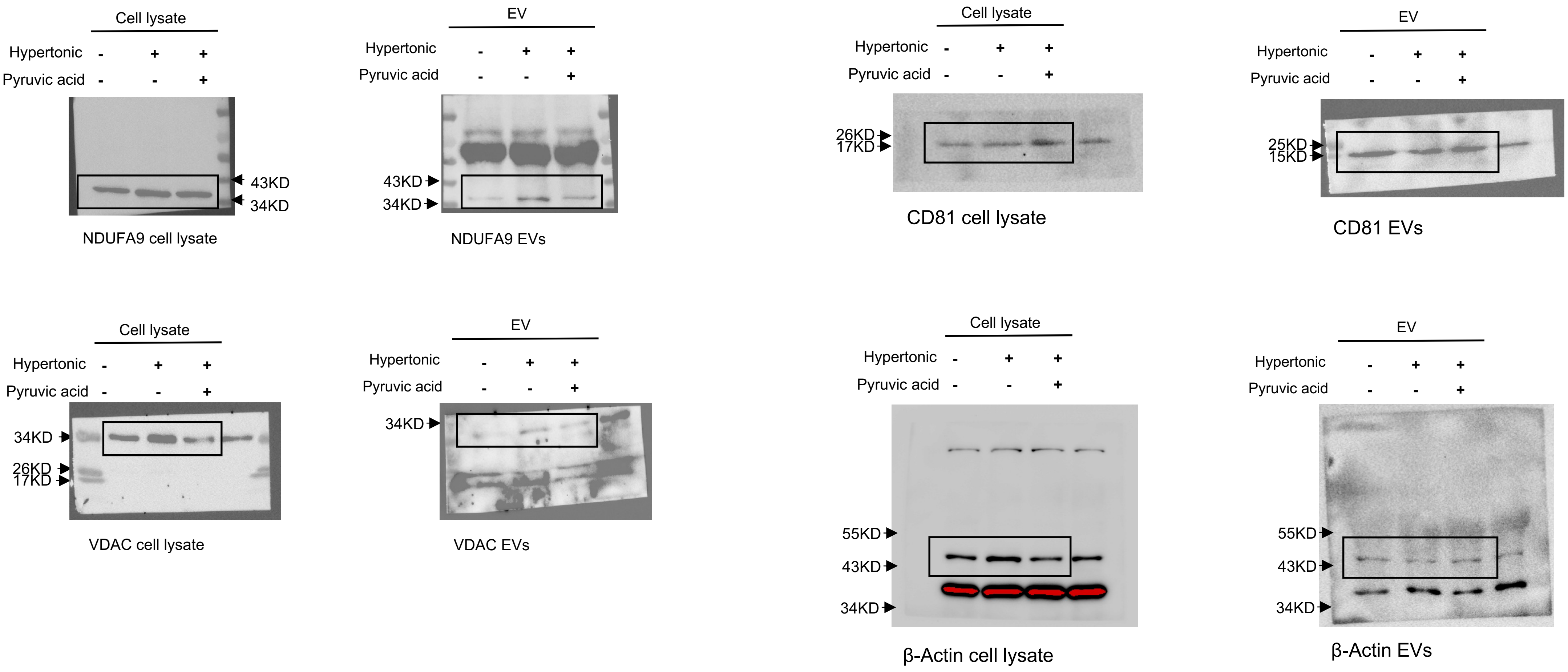

Additional file 1.

Original western blot gels of Fig. 2H for cell lysates and EVs of 3T3-L1 adipocytes following indicated treatments were subject to immunoblot analyses of the indicated proteins, respectively.

Figure 4B

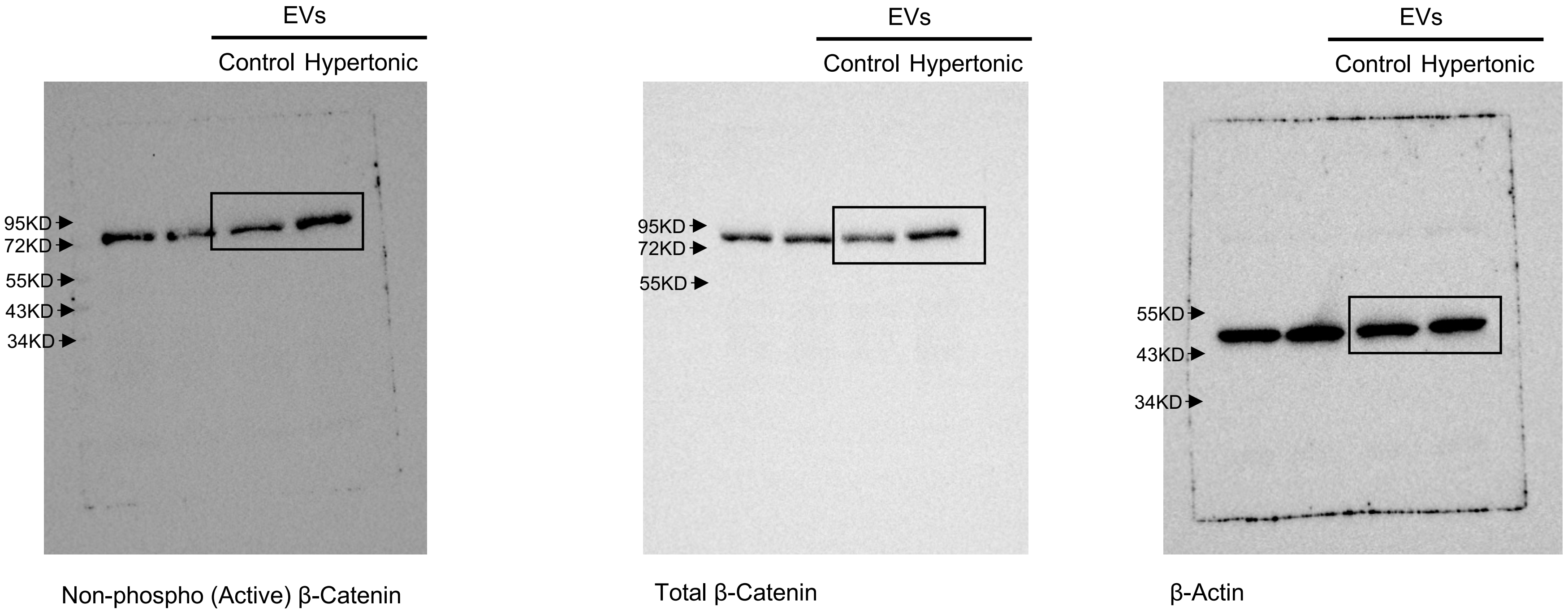

Additional file 2.

Original western blot gels of Fig. 4B for total cell lysates from 3T3-L1 adipocytes treated by EVs from isotonic or hypertonic adipocyte cultures were subject to western blot analyses of indicated proteins.

Figure 4C

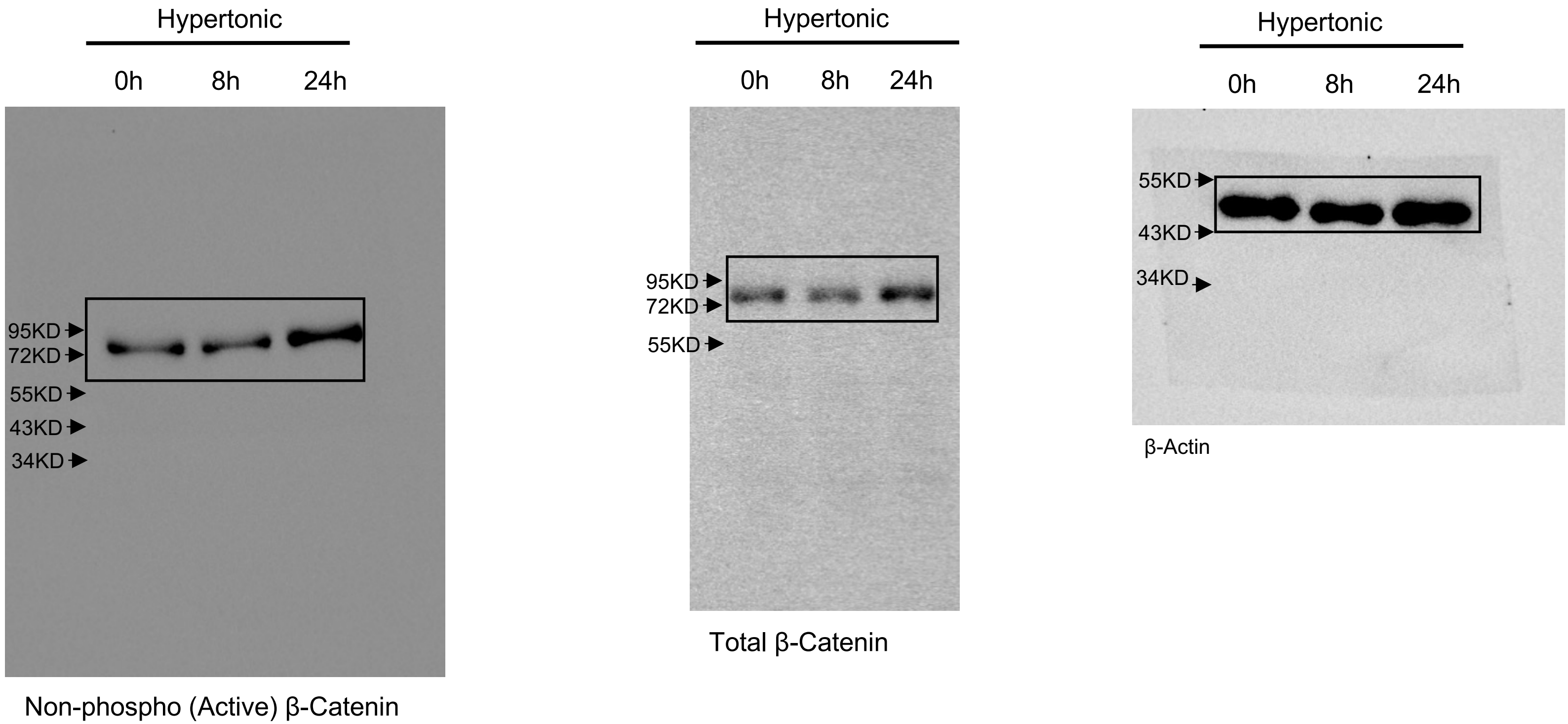

Additional file 3.

Original western blot gels of Fig. 4C for total cell lysates from 3T3-L1 adipocytes in indicated culture conditions were subject to western blot analyses of indicated proteins.

Figure 4F

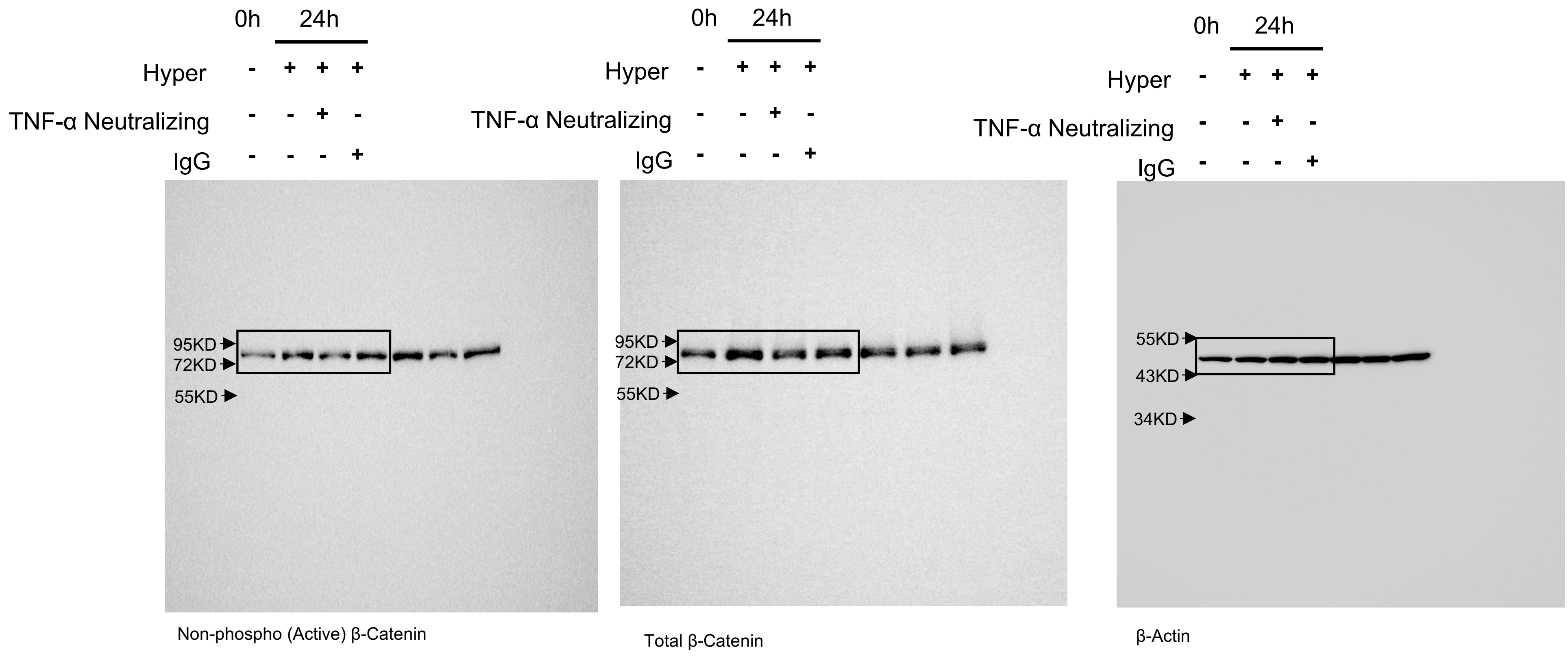

<u>Additional file 4.</u>

Original western blot gels of Fig. 4F for 3T3-L1 adipocytes were treated with the indicated conditions for 24 hours and the cell lysates were subject to western blot analysis of indicated proteins.

Supplementary Fig. 2.

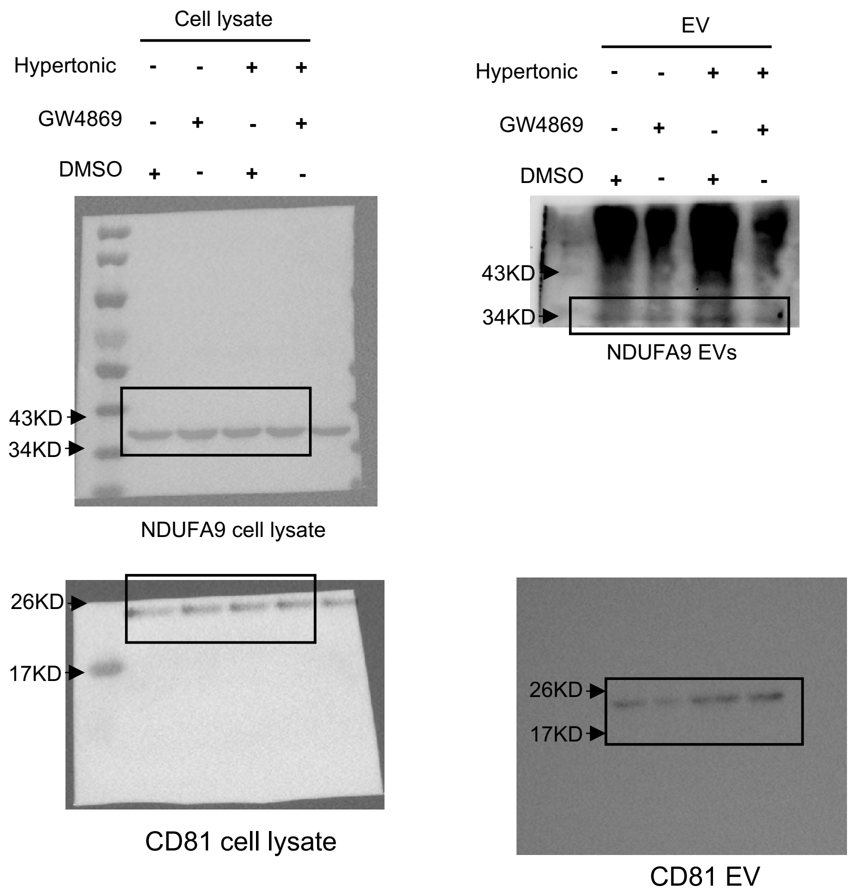

Additional file 5.

Original western blot gels of supplementary Fig. 4. for 3T3-L1 adipocytes were treated with the indicated conditions and the cell lysates and EVs were subject to western blot analysis of indicated proteins
