# Supplementary material for: Hypertonicity induces mitochondrial extracellular vesicles (MEVs) that activate TNF-α and β-catenin signaling to promote adipocyte dedifferentiation
Source: Stem Cell Res Ther. 2023 Dec 20;14:333. doi: 10.1186/s13287-023-03558-3 (PMC10731851; doi:10.1186/s13287-023-03558-3)
Supplement: Supplementary file 2 — Additional file 2. Supplementary Figures and Tables. [file 13287_2023_3558_MOESM2_ESM.docx]

**Hypertonicity induces mitochondrial extracellular vesicles**

**(MEVs)that activate TNF-α and β-catenin signalling to**

**promote adipocyte dedifferentiation**

Guopan Liu^1,2^, Ying Wang^1,2^, Yilin Pan^1,2^, Li Tian^1,2^, Ming Ho Choi^1^, Li Wang^1^,

Jin Young Kim^1^, Jian Zhang^3^, Shuk Han Cheng^1^, Liang Zhang^1,2^*

^1^Department of Biomedical Sciences, College of Veterinary Medicine and Life Sciences, City University of Hong Kong, 83 Tat Chee Avenue, Kowloon, Hong Kong, China.

^2^Key Laboratory of Biochip Technology, Biotech and Health Centre, Shenzhen Research Institute of City University of Hong Kong, Shenzhen, 518057, China.

^3^Medicinal Chemistry and Bioinformatics Center, Shanghai Jiao Tong University, School of Medicine, Shanghai 200025, China.

*Address correspondence to [liangzhang.28@cityu.edu.hk](mailto:liangzhang.28@cityu.edu.hk)

**Table of content**

1. Supplementary Methods3

1.1 Immuno/fluorescent labeling and microscopy3

1.2 qPCR gene expression analysis3

2. Supplementary Figures4

2.1 Supplementary Figure 14

2.2 Supplementary Figure 25

2.3 Supplementary Figure 36

2.4 Supplementary Figure 47

3. Supplementary Table7

2.1 Supplementary Table 17

2.2 Supplementary Table 28

2.3 Supplementary Table 39

**Supplementary Methods**

**Immuno/fluorescent labeling and microscopy**

Twelve hours before the experiment, cells were seeded onto cover slips in 12-well dishes and cultured overnight at 37°C and 5% CO_2_. Cells were fixed with 2% paraformaldehyde/PBS for 10 min at 4°C. Next, the cells were either directly incubated with 1% BSA/PBS for 1 h or first permeabilized with 0.1% Triton X-100/PBS for 10 min at 4°C before blocking with 1% BSA/PBS. Subsequently, CD13 primary antibody (Santa Cruz, product no. sc-13536 1:100) or Endoglin primary antibody (Santa Cruz, product no. sc-18838 1:100) was incubated with cells overnight at 4°C. Following 3 times washing in 0.5% tween/PBS, cells were incubated with secondary antibody (CST, product no.8890S) in 1% BSA/PBS (1:400) in darkness for 1.5 hours at room temperature. After further washing, cells were mounted with DAPI (Thermo Fisher, product no. 62247) and examined with a Nikon A1HD25 confocal microscope.

**qPCR gene expression analysis**

Total RNA was isolated using RNA Extraction Kit (Takara,product no. 9767) according to the manufacturer's recommended protocol. First-strand cDNA was synthesized using the PrimeScript RT Reagent Kit(Takara, product no. RR047A) for RT-PCR. Mix 2 μl of 5× PrimeScript RT Master Mix (Perfect Real Time) with RNase-Free Distilled Water and RNA solution until the total amount of RNA was 500 ng in a 10 μl system. Transferred the reaction solution to ABI ProFlex PCR System (2 × 96-well) to perform reverse transcription. RT-PCR was performed using the Ex-Taq PCR kit (Takara, product no. RR820A) according to the manufacturer's instructions.

**
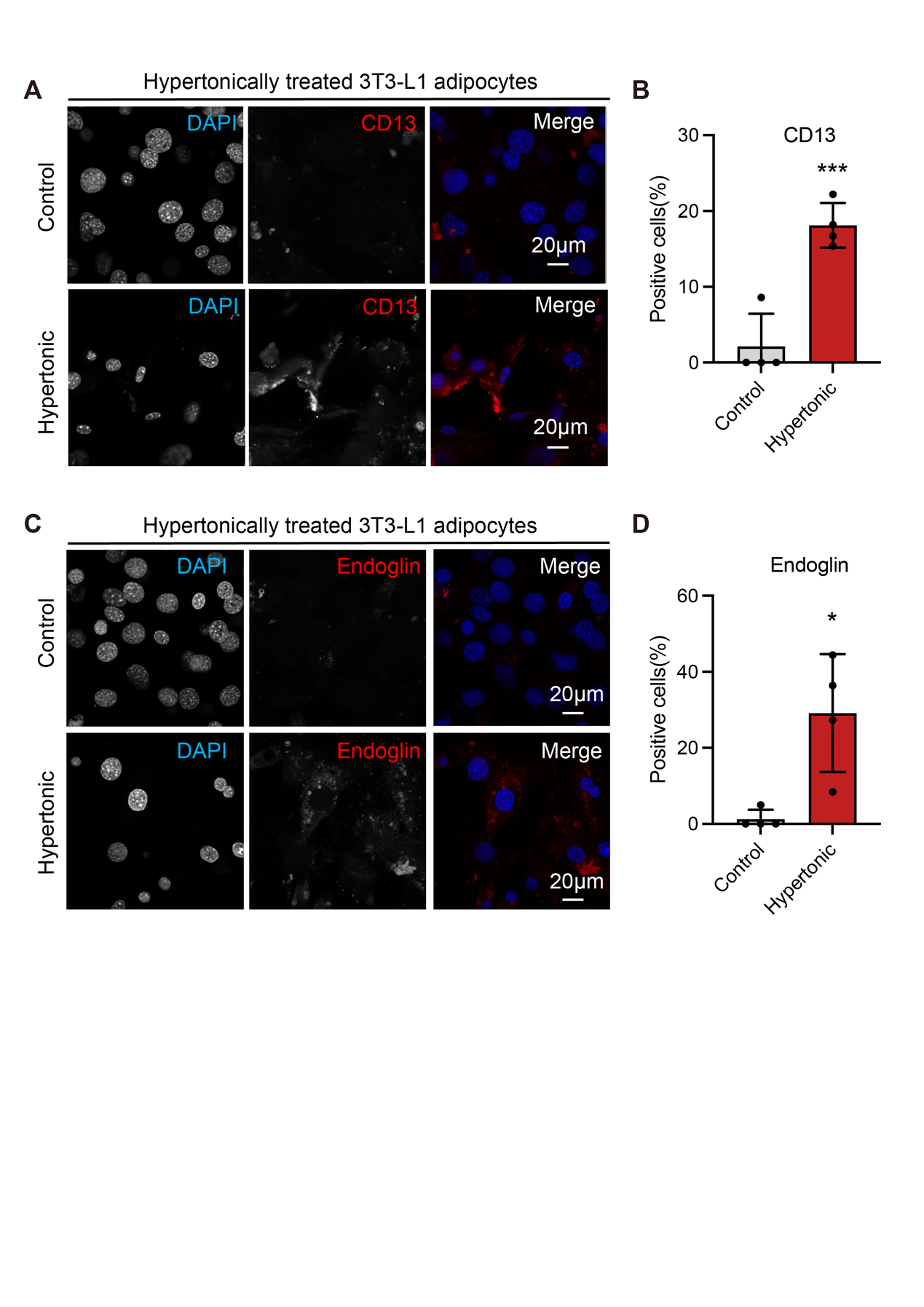
**

**Supplementary Fig. 1. The surface markers of dedifferentiated adipocytes induce by hypertonic treatment. (A-B)** Immunostaining of CD13 in control and hypertonic treated 3T3-L1 adipocytes. Percentage of cells with positive CD13 labeling are quantified in (B) Scale bar: 20 µm. **(C-D)** Immunostaining of Endoglin in control and hypertonic treated 3T3-L1 adipocytes. Percentage of cells with positive Endoglin labeling are quantified in (D) Scale bar: 20 µm.


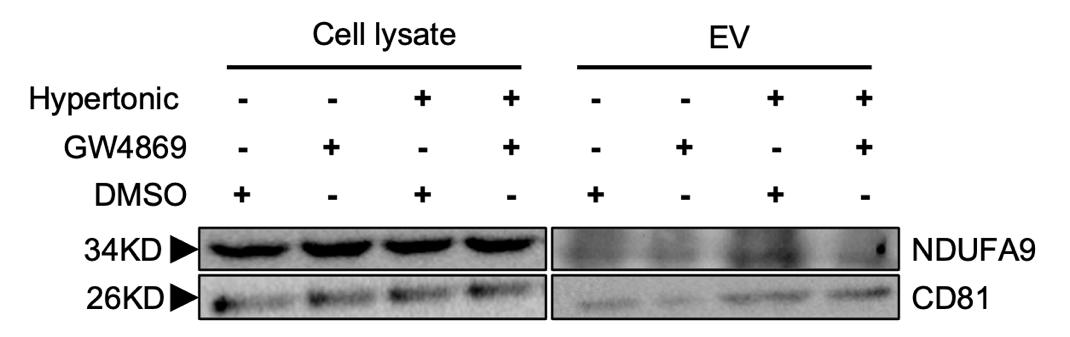


**Supplementary Fig. 2.** The levels of NDUFA9 and CD81 were assessed by Western blot analysis in 3T3-L1 adipocytes after the indicated treatments, including isotonic+DMSO, isotonic+GW4869, hypertonic+DMSO, or hypertonic+GW4869. Full-length blots are presented in Additional file 5.

**
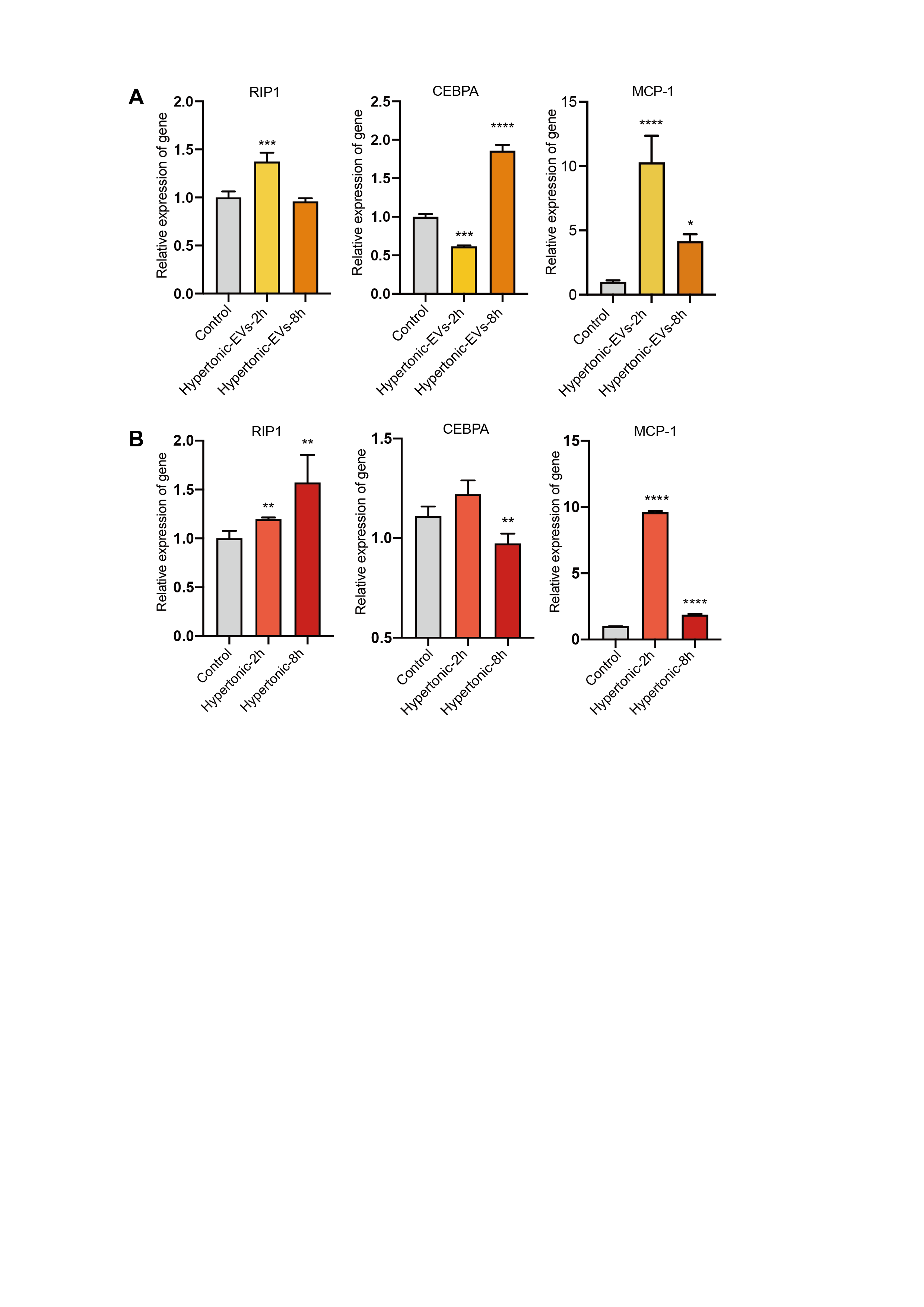
**

**Supplementary Fig. 3. Hypertonicity and mitochondrial EVs (MEVs) activates TNF-α signaling in 3T3-L1 adipocytes. (A)** RT-qPCR analysis of the expression of RIP1, CEBPA and MCP-1 in 3T3-L1 adipocytes treated with EVs isolated from the adipocytes from the indicated cultures. **(B)** RT-qPCR analysis of the expression of RIP1, CEBPA and MCP-1 in 3T3-L1 adipocytes receiving indicated treatments.


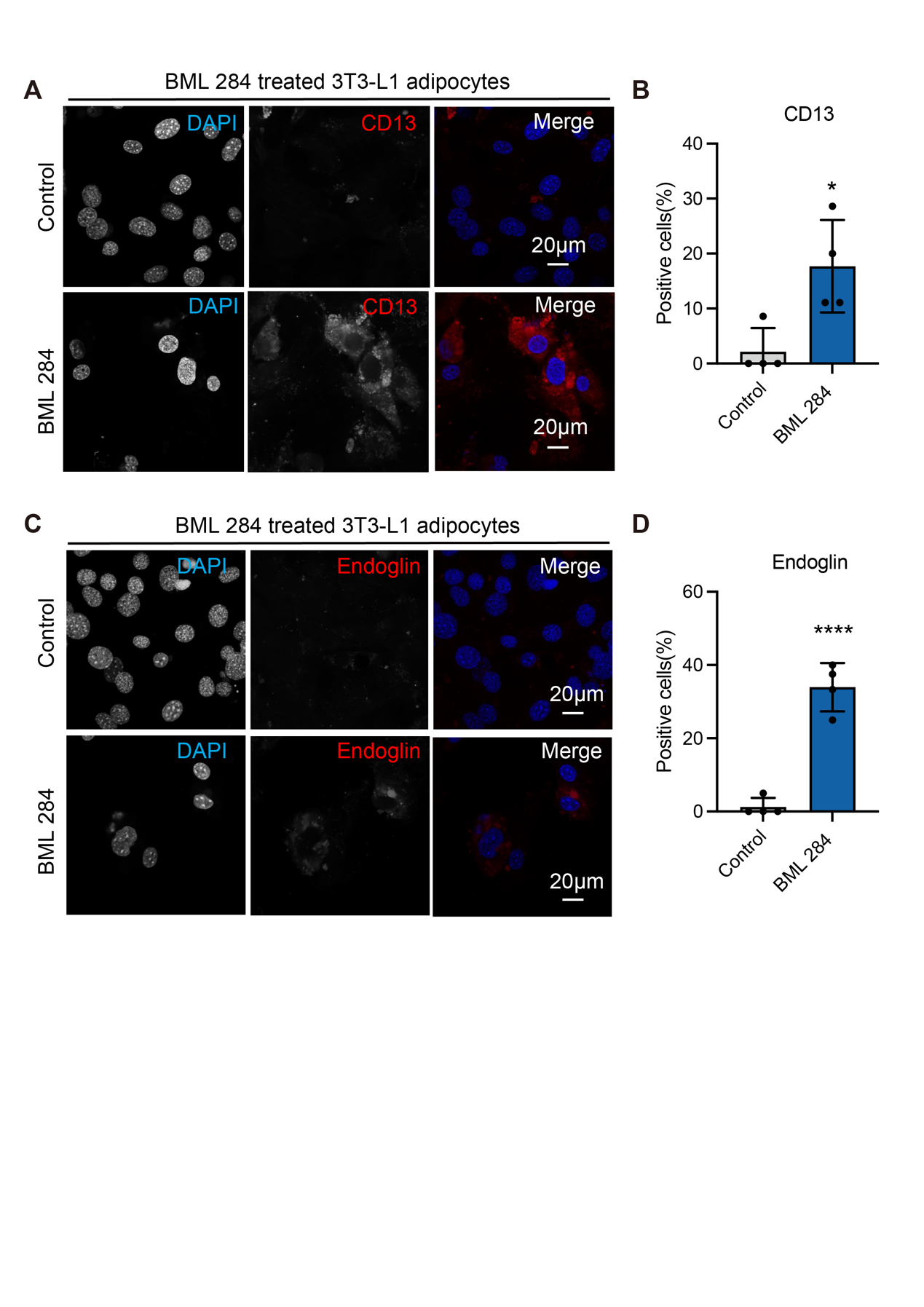


**Supplementary Fig. 4. The surface markers of dedifferentiated adipocytes induce by BML 284. (A-B)** Immunostaining of CD13 in control and BML 284 treated 3T3-L1 adipocytes. Percentage of cells with positive CD13 labeling are quantified in (B) Scale bar: 20 µm. **(C-D)** Immunostaining of Endoglin in control and BML 284 treated 3T3-L1 adipocytes. Percentage of cells with positive Endoglin labeling are quantified in (D) Scale bar: 20 µm.

**Supplementary Table**

| **Table S1. List of primer sequences used for qPCR validation** | | |
| --- | --- | --- |
| Primers qRT-PCR | Forward Primer 5′-3′ | Reverse primer 5′-3′ |
| 18SRNA | CTGAGAAACGGCTACCACATC | GCCTCGAAAGAGTCCTGTATTG |
| TNF-α | ATGGCCTCCCTCTCATCAGT | CTTGGTGGTTTGCTACGACG |
| RIP1 | CACTGCGATCATTCTCGTCCTG | GACTGTGTACCCTTACCTCCGA |
| IL-6 | GTTCTCTGGGAAATCGTGGA | GCATTGGAAATTGGGGTAGG |
| MCP-1 | AGCCAACTCTCACTGAAGCC | GGACCCATTCCTTCTTGGGG |
| Col2al | CCGCAGTCACTCCAGGAT | TGCAGTCTGCCCAGTTCA |
| Sox9 | AGCTCACCAGACCCTGAGAA | TCCCAGCAATCGTTACCTTC |
| Smad3 | GTCAACAAGTGGTGGCGTGTG | GCAGCAAAGGCTTCTGGGATAA |
| Esrrb | GTGCGCAGGTACAAGAAACT | CCAGGTTCTCAATGTACATCGA |
| Sox2 | GCGGAGTGGAAACTTTTGTCC | GGGAAGCGTGTACTTATCCTTCT |
| Smad9 | CGGATGAGCTTTGTGAAGG | GGGTGCTCGTGACATCCT |
| Runc2 | GCCGGGAATGATGAGAACTA | GGTGAAACTCTTGCCTCGTC |
| ALP | TGAGCGACACGGACAAGA | GGCCTGGTAGTTGTTGTGAG |

**Supplementary Table 1.**

List of primer sequences used for qPCR validation

| **Table S2. The most represented GO terms of cellular component of hypertonic unique proteins** | | | |
| --- | --- | --- | --- |
| Gene Ontology | Cellular Component | | |
| No | GOID | Description | FDR |
| 1 | GO:0005743 | Mitochondrial inner membrane | 1.03E-02 |
| 2 | GO:0005739 | Mitochondrion | 3.16E-02 |
| 3 | GO:0031967 | Organelle envelope | 4.29E-02 |

**Supplementary Table 2.**

The most represented GO terms of cellular components of unique proteins from hypertonic EVs

| **Table S3. The fold change between control and hypertonic group** | | | | | | |
| --- | --- | --- | --- | --- | --- | --- |
| Accession | Control (Normalized abundance) | Hypertonic (Normalized abundance) | lg(control) | lg(hypertonic) | lg Fold change | Group |
| Q6NSP9 | 8584594.5 | 1000 | 6.933719786 | 3 | -3.933719786 | Proteins of >= 10-fold downregulation in hypertonic EVs |
| P14131 | 7320493.5 | 1000 | 6.864540359 | 3 | -3.864540359 | Proteins of >= 10-fold downregulation in hypertonic EVs |
| P25444 | 6898404.625 | 1000 | 6.838748664 | 3 | -3.838748664 | Proteins of >= 10-fold downregulation in hypertonic EVs |
| Q8C266 | 5759487.5 | 1000 | 6.76038384 | 3 | -3.76038384 | Proteins of >= 10-fold downregulation in hypertonic EVs |
| Q8VED5 | 5167437 | 1000 | 6.713275191 | 3 | -3.713275191 | Proteins of >= 10-fold downregulation in hypertonic EVs |
| A0A1L1SV25 | 5002853 | 1000 | 6.699217742 | 3 | -3.699217742 | Proteins of >= 10-fold downregulation in hypertonic EVs |
| A0A140T8M7 | 4811706.875 | 1000 | 6.682299163 | 3 | -3.682299163 | Proteins of >= 10-fold downregulation in hypertonic EVs |
| P68040 | 3897300.25 | 1000 | 6.590763865 | 3 | -3.590763865 | Proteins of >= 10-fold downregulation in hypertonic EVs |
| P19324 | 3806003.5 | 1000 | 6.580469183 | 3 | -3.580469183 | Proteins of >= 10-fold downregulation in hypertonic EVs |
| P84104 | 3514547.5 | 1000 | 6.545869417 | 3 | -3.545869417 | Proteins of >= 10-fold downregulation in hypertonic EVs |
| P62320 | 3417735.25 | 1000 | 6.533738418 | 3 | -3.533738418 | Proteins of >= 10-fold downregulation in hypertonic EVs |
| Q922Q8 | 3086186.125 | 1000 | 6.489422114 | 3 | -3.489422114 | Proteins of >= 10-fold downregulation in hypertonic EVs |
| P54116 | 3019815 | 1000 | 6.479980338 | 3 | -3.479980338 | Proteins of >= 10-fold downregulation in hypertonic EVs |
| Q9WTR5 | 2909470.75 | 1000 | 6.463813995 | 3 | -3.463813995 | Proteins of >= 10-fold downregulation in hypertonic EVs |
| P62301 | 2566389 | 1000 | 6.409322485 | 3 | -3.409322485 | Proteins of >= 10-fold downregulation in hypertonic EVs |
| P32067 | 2485558 | 1000 | 6.395423902 | 3 | -3.395423902 | Proteins of >= 10-fold downregulation in hypertonic EVs |
| Q9QXS1 | 2323585.563 | 1000 | 6.366158669 | 3 | -3.366158669 | Proteins of >= 10-fold downregulation in hypertonic EVs |
| Q9DCX2 | 2290584.563 | 1000 | 6.35994633 | 3 | -3.35994633 | Proteins of >= 10-fold downregulation in hypertonic EVs |
| A0A0R4J1E2 | 2246978 | 1000 | 6.35159882 | 3 | -3.35159882 | Proteins of >= 10-fold downregulation in hypertonic EVs |
| Q6NXH9 | 2201763 | 1000 | 6.342770569 | 3 | -3.342770569 | Proteins of >= 10-fold downregulation in hypertonic EVs |
| P80316 | 1914071.625 | 1000 | 6.281958185 | 3 | -3.281958185 | Proteins of >= 10-fold downregulation in hypertonic EVs |
| Q9CYL5 | 1717588 | 1000 | 6.234918997 | 3 | -3.234918997 | Proteins of >= 10-fold downregulation in hypertonic EVs |
| P67984 | 1695660.719 | 1000 | 6.22933896 | 3 | -3.22933896 | Proteins of >= 10-fold downregulation in hypertonic EVs |
| F8WID5 | 1633021 | 1000 | 6.21299177 | 3 | -3.21299177 | Proteins of >= 10-fold downregulation in hypertonic EVs |
| P61358 | 1606448.875 | 1000 | 6.205866909 | 3 | -3.205866909 | Proteins of >= 10-fold downregulation in hypertonic EVs |
| Q9CQ22 | 1604461.875 | 1000 | 6.205329402 | 3 | -3.205329402 | Proteins of >= 10-fold downregulation in hypertonic EVs |
| A0A0R4J0I9 | 1534512.875 | 1000 | 6.185970537 | 3 | -3.185970537 | Proteins of >= 10-fold downregulation in hypertonic EVs |
| P47753 | 1521776.375 | 1000 | 6.182350838 | 3 | -3.182350838 | Proteins of >= 10-fold downregulation in hypertonic EVs |
| Q3TLP8 | 1442923.625 | 1000 | 6.159243344 | 3 | -3.159243344 | Proteins of >= 10-fold downregulation in hypertonic EVs |
| F8WGL3 | 1404188 | 1000 | 6.147425257 | 3 | -3.147425257 | Proteins of >= 10-fold downregulation in hypertonic EVs |
| Q9WVJ3 | 1375430.25 | 1000 | 6.138438572 | 3 | -3.138438572 | Proteins of >= 10-fold downregulation in hypertonic EVs |
| P06801 | 1335398.75 | 1000 | 6.125610965 | 3 | -3.125610965 | Proteins of >= 10-fold downregulation in hypertonic EVs |
| P12815 | 1254106.25 | 1000 | 6.098334332 | 3 | -3.098334332 | Proteins of >= 10-fold downregulation in hypertonic EVs |
| G5E902 | 1184277.125 | 1000 | 6.073453341 | 3 | -3.073453341 | Proteins of >= 10-fold downregulation in hypertonic EVs |
| P59108 | 1112840.75 | 1000 | 6.04643302 | 3 | -3.04643302 | Proteins of >= 10-fold downregulation in hypertonic EVs |
| P80315 | 1074040.188 | 1000 | 6.031020532 | 3 | -3.031020532 | Proteins of >= 10-fold downregulation in hypertonic EVs |
| Q9D0F3 | 1057436.344 | 1000 | 6.024254233 | 3 | -3.024254233 | Proteins of >= 10-fold downregulation in hypertonic EVs |
| P62835 | 1017386.375 | 1000 | 6.007485917 | 3 | -3.007485917 | Proteins of >= 10-fold downregulation in hypertonic EVs |
| P42669 | 994102.625 | 1000 | 5.997431221 | 3 | -2.997431221 | Proteins of >= 10-fold downregulation in hypertonic EVs |
| Q6R0H7 | 973654.875 | 1000 | 5.988405043 | 3 | -2.988405043 | Proteins of >= 10-fold downregulation in hypertonic EVs |
| Q640N1 | 972075.3125 | 1000 | 5.987699914 | 3 | -2.987699914 | Proteins of >= 10-fold downregulation in hypertonic EVs |
| Q9Z1G4 | 960960 | 1000 | 5.982705311 | 3 | -2.982705311 | Proteins of >= 10-fold downregulation in hypertonic EVs |
| Q7TMM9 | 927458.875 | 1000 | 5.967294661 | 3 | -2.967294661 | Proteins of >= 10-fold downregulation in hypertonic EVs |
| A2AE89 | 910184.1875 | 1000 | 5.959129286 | 3 | -2.959129286 | Proteins of >= 10-fold downregulation in hypertonic EVs |
| O08547 | 899735.8125 | 1000 | 5.954115007 | 3 | -2.954115007 | Proteins of >= 10-fold downregulation in hypertonic EVs |
| P50428 | 870805.5 | 1000 | 5.939921163 | 3 | -2.939921163 | Proteins of >= 10-fold downregulation in hypertonic EVs |
| Q8VDN2 | 857618.1094 | 1000 | 5.933293943 | 3 | -2.933293943 | Proteins of >= 10-fold downregulation in hypertonic EVs |
| A0A1L1SQU7 | 828608.9375 | 1000 | 5.918349613 | 3 | -2.918349613 | Proteins of >= 10-fold downregulation in hypertonic EVs |
| Q9JM62 | 812571.4375 | 1000 | 5.909861552 | 3 | -2.909861552 | Proteins of >= 10-fold downregulation in hypertonic EVs |
| O09061 | 810664.5 | 1000 | 5.908841155 | 3 | -2.908841155 | Proteins of >= 10-fold downregulation in hypertonic EVs |
| P09103 | 809642.625 | 1000 | 5.908293364 | 3 | -2.908293364 | Proteins of >= 10-fold downregulation in hypertonic EVs |
| Q8BL97 | 803354.5625 | 1000 | 5.904907265 | 3 | -2.904907265 | Proteins of >= 10-fold downregulation in hypertonic EVs |
| P15532 | 771185.5 | 1000 | 5.887158855 | 3 | -2.887158855 | Proteins of >= 10-fold downregulation in hypertonic EVs |
| O54965 | 716947.9375 | 1000 | 5.85548762 | 3 | -2.85548762 | Proteins of >= 10-fold downregulation in hypertonic EVs |
| O08992 | 709786.5 | 1000 | 5.851127735 | 3 | -2.851127735 | Proteins of >= 10-fold downregulation in hypertonic EVs |
| Q62318 | 700194.4375 | 1000 | 5.845218656 | 3 | -2.845218656 | Proteins of >= 10-fold downregulation in hypertonic EVs |
| P08207 | 699769 | 1000 | 5.844954699 | 3 | -2.844954699 | Proteins of >= 10-fold downregulation in hypertonic EVs |
| P16460 | 697613.75 | 1000 | 5.843615032 | 3 | -2.843615032 | Proteins of >= 10-fold downregulation in hypertonic EVs |
| Q921F2 | 696275.6875 | 1000 | 5.842781231 | 3 | -2.842781231 | Proteins of >= 10-fold downregulation in hypertonic EVs |
| E9QP00 | 694917.375 | 1000 | 5.84193317 | 3 | -2.84193317 | Proteins of >= 10-fold downregulation in hypertonic EVs |
| P63325 | 680642.0625 | 1000 | 5.832918784 | 3 | -2.832918784 | Proteins of >= 10-fold downregulation in hypertonic EVs |
| P97384 | 660499.9375 | 1000 | 5.819872781 | 3 | -2.819872781 | Proteins of >= 10-fold downregulation in hypertonic EVs |
| Q8CI43 | 655367.25 | 1000 | 5.816484735 | 3 | -2.816484735 | Proteins of >= 10-fold downregulation in hypertonic EVs |
| P05201 | 638063 | 1000 | 5.804863561 | 3 | -2.804863561 | Proteins of >= 10-fold downregulation in hypertonic EVs |
| E9Q1G8 | 627734.4375 | 1000 | 5.797775955 | 3 | -2.797775955 | Proteins of >= 10-fold downregulation in hypertonic EVs |
| Q8C166 | 618277.25 | 1000 | 5.791183267 | 3 | -2.791183267 | Proteins of >= 10-fold downregulation in hypertonic EVs |
| Q3TWW8 | 607454.6875 | 1000 | 5.783513888 | 3 | -2.783513888 | Proteins of >= 10-fold downregulation in hypertonic EVs |
| O70378 | 605887.9375 | 1000 | 5.782392306 | 3 | -2.782392306 | Proteins of >= 10-fold downregulation in hypertonic EVs |
| O08749 | 589564.5 | 1000 | 5.770531325 | 3 | -2.770531325 | Proteins of >= 10-fold downregulation in hypertonic EVs |
| Q3TW96 | 581283.5625 | 1000 | 5.764388042 | 3 | -2.764388042 | Proteins of >= 10-fold downregulation in hypertonic EVs |
| O55029 | 558075.0625 | 1000 | 5.746692617 | 3 | -2.746692617 | Proteins of >= 10-fold downregulation in hypertonic EVs |
| P37804 | 546013.5625 | 1000 | 5.73720343 | 3 | -2.73720343 | Proteins of >= 10-fold downregulation in hypertonic EVs |
| Q9R0P5 | 532583.4375 | 1000 | 5.726387656 | 3 | -2.726387656 | Proteins of >= 10-fold downregulation in hypertonic EVs |
| E9PY39 | 521401.5938 | 1000 | 5.717172354 | 3 | -2.717172354 | Proteins of >= 10-fold downregulation in hypertonic EVs |
| Q9R1Q7 | 509098.8438 | 1000 | 5.706802111 | 3 | -2.706802111 | Proteins of >= 10-fold downregulation in hypertonic EVs |
| A0A140T8T4 | 508990.0938 | 1000 | 5.70670933 | 3 | -2.70670933 | Proteins of >= 10-fold downregulation in hypertonic EVs |
| P60335 | 505058.4063 | 1000 | 5.703341604 | 3 | -2.703341604 | Proteins of >= 10-fold downregulation in hypertonic EVs |
| Q8BML9 | 499788.4375 | 1000 | 5.698786205 | 3 | -2.698786205 | Proteins of >= 10-fold downregulation in hypertonic EVs |
| H7BX01 | 494756.7188 | 1000 | 5.694391701 | 3 | -2.694391701 | Proteins of >= 10-fold downregulation in hypertonic EVs |
| P01029 | 490821.5313 | 1000 | 5.690923606 | 3 | -2.690923606 | Proteins of >= 10-fold downregulation in hypertonic EVs |
| P35550 | 473295.1875 | 1000 | 5.675132089 | 3 | -2.675132089 | Proteins of >= 10-fold downregulation in hypertonic EVs |
| Q8BGQ7 | 472780.8125 | 1000 | 5.674659843 | 3 | -2.674659843 | Proteins of >= 10-fold downregulation in hypertonic EVs |
| P08122 | 445774.125 | 1000 | 5.649114856 | 3 | -2.649114856 | Proteins of >= 10-fold downregulation in hypertonic EVs |
| A0A087WQW8 | 444687.1563 | 1000 | 5.648054586 | 3 | -2.648054586 | Proteins of >= 10-fold downregulation in hypertonic EVs |
| P42932 | 439441 | 1000 | 5.642900574 | 3 | -2.642900574 | Proteins of >= 10-fold downregulation in hypertonic EVs |
| Q9JIK5 | 428586.1875 | 1000 | 5.63203817 | 3 | -2.63203817 | Proteins of >= 10-fold downregulation in hypertonic EVs |
| D3Z722 | 414901.5313 | 1000 | 5.617945038 | 3 | -2.617945038 | Proteins of >= 10-fold downregulation in hypertonic EVs |
| Q8BG05 | 412027.5 | 1000 | 5.614926203 | 3 | -2.614926203 | Proteins of >= 10-fold downregulation in hypertonic EVs |
| E9Q3E1 | 411143.5938 | 1000 | 5.613993528 | 3 | -2.613993528 | Proteins of >= 10-fold downregulation in hypertonic EVs |
| P28654 | 407931.5938 | 1000 | 5.610587342 | 3 | -2.610587342 | Proteins of >= 10-fold downregulation in hypertonic EVs |
| P62827 | 403111.9063 | 1000 | 5.605425626 | 3 | -2.605425626 | Proteins of >= 10-fold downregulation in hypertonic EVs |
| Q91VH2 | 394732.75 | 1000 | 5.59630316 | 3 | -2.59630316 | Proteins of >= 10-fold downregulation in hypertonic EVs |
| Q8K4Z5 | 391994.1875 | 1000 | 5.593279627 | 3 | -2.593279627 | Proteins of >= 10-fold downregulation in hypertonic EVs |
| Q9CQV8 | 369977.6563 | 1000 | 5.568175497 | 3 | -2.568175497 | Proteins of >= 10-fold downregulation in hypertonic EVs |
| Q62167 | 360194.0938 | 1000 | 5.556536587 | 3 | -2.556536587 | Proteins of >= 10-fold downregulation in hypertonic EVs |
| P63163 | 359407.0625 | 1000 | 5.555586607 | 3 | -2.555586607 | Proteins of >= 10-fold downregulation in hypertonic EVs |
| Q9Z1E4 | 352884.6563 | 1000 | 5.547632775 | 3 | -2.547632775 | Proteins of >= 10-fold downregulation in hypertonic EVs |
| E0CXB9 | 333323.9063 | 1000 | 5.522866463 | 3 | -2.522866463 | Proteins of >= 10-fold downregulation in hypertonic EVs |
| E9Q557 | 328572.5313 | 1000 | 5.516631254 | 3 | -2.516631254 | Proteins of >= 10-fold downregulation in hypertonic EVs |
| P11983 | 324146.4375 | 1000 | 5.510741253 | 3 | -2.510741253 | Proteins of >= 10-fold downregulation in hypertonic EVs |
| F8VPU2 | 323206.375 | 1000 | 5.509479918 | 3 | -2.509479918 | Proteins of >= 10-fold downregulation in hypertonic EVs |
| O54734 | 309466.2813 | 1000 | 5.490613336 | 3 | -2.490613336 | Proteins of >= 10-fold downregulation in hypertonic EVs |
| P14901 | 304527.7813 | 1000 | 5.483626918 | 3 | -2.483626918 | Proteins of >= 10-fold downregulation in hypertonic EVs |
| Q3UH59 | 301750.3125 | 1000 | 5.479647729 | 3 | -2.479647729 | Proteins of >= 10-fold downregulation in hypertonic EVs |
| P63101 | 296965.25 | 1000 | 5.472705632 | 3 | -2.472705632 | Proteins of >= 10-fold downregulation in hypertonic EVs |
| Q9EST5 | 293687.8438 | 1000 | 5.467885971 | 3 | -2.467885971 | Proteins of >= 10-fold downregulation in hypertonic EVs |
| Q00612 | 292937.0625 | 1000 | 5.466774322 | 3 | -2.466774322 | Proteins of >= 10-fold downregulation in hypertonic EVs |
| Q9WVA4 | 291106.4375 | 1000 | 5.464051809 | 3 | -2.464051809 | Proteins of >= 10-fold downregulation in hypertonic EVs |
| Q91V41 | 288433 | 1000 | 5.460044947 | 3 | -2.460044947 | Proteins of >= 10-fold downregulation in hypertonic EVs |
| P18872 | 284469.4688 | 1000 | 5.454035662 | 3 | -2.454035662 | Proteins of >= 10-fold downregulation in hypertonic EVs |
| Q8BH95 | 269171.9063 | 1000 | 5.43002973 | 3 | -2.43002973 | Proteins of >= 10-fold downregulation in hypertonic EVs |
| Q9ESX5 | 260158.1563 | 1000 | 5.415237446 | 3 | -2.415237446 | Proteins of >= 10-fold downregulation in hypertonic EVs |
| P45376 | 232747.25 | 1000 | 5.366884558 | 3 | -2.366884558 | Proteins of >= 10-fold downregulation in hypertonic EVs |
| P57716 | 232310.5781 | 1000 | 5.366068986 | 3 | -2.366068986 | Proteins of >= 10-fold downregulation in hypertonic EVs |
| Q8K2Y3 | 224756.2344 | 1000 | 5.351711747 | 3 | -2.351711747 | Proteins of >= 10-fold downregulation in hypertonic EVs |
| P80314 | 215186.7656 | 1000 | 5.332815558 | 3 | -2.332815558 | Proteins of >= 10-fold downregulation in hypertonic EVs |
| Q80X90 | 203321.0469 | 1000 | 5.308182337 | 3 | -2.308182337 | Proteins of >= 10-fold downregulation in hypertonic EVs |
| Q91VD9 | 202838.5469 | 1000 | 5.307150491 | 3 | -2.307150491 | Proteins of >= 10-fold downregulation in hypertonic EVs |
| Q9JHU4 | 188149.375 | 1000 | 5.27450278 | 3 | -2.27450278 | Proteins of >= 10-fold downregulation in hypertonic EVs |
| P10922 | 83964977.31 | 450128.0052 | 7.924098175 | 5.653336034 | -2.270762141 | Proteins of >= 10-fold downregulation in hypertonic EVs |
| Q9Z1P8 | 186305.0313 | 1000 | 5.270224583 | 3 | -2.270224583 | Proteins of >= 10-fold downregulation in hypertonic EVs |
| J3QNK8 | 173735.8281 | 1000 | 5.239889389 | 3 | -2.239889389 | Proteins of >= 10-fold downregulation in hypertonic EVs |
| Q8CGK3 | 169412.4063 | 1000 | 5.228945211 | 3 | -2.228945211 | Proteins of >= 10-fold downregulation in hypertonic EVs |
| F8WHL2 | 155302.9844 | 1000 | 5.191179801 | 3 | -2.191179801 | Proteins of >= 10-fold downregulation in hypertonic EVs |
| P17809 | 145815.9844 | 1000 | 5.163805134 | 3 | -2.163805134 | Proteins of >= 10-fold downregulation in hypertonic EVs |
| Q91VE0 | 134064.8906 | 1000 | 5.127315058 | 3 | -2.127315058 | Proteins of >= 10-fold downregulation in hypertonic EVs |
| P15920 | 115438.2422 | 1000 | 5.062349705 | 3 | -2.062349705 | Proteins of >= 10-fold downregulation in hypertonic EVs |
| Q3U3J1 | 104937.3359 | 1000 | 5.020930034 | 3 | -2.020930034 | Proteins of >= 10-fold downregulation in hypertonic EVs |
| F2Z3U4 | 66911.48438 | 1000 | 4.825500664 | 3 | -1.825500664 | Proteins of >= 10-fold downregulation in hypertonic EVs |
| P43276 | 463790690.3 | 7201846.276 | 8.666322027 | 6.857443847 | -1.80887818 | Proteins of >= 10-fold downregulation in hypertonic EVs |
| Q9CY66 | 53491.60156 | 1000 | 4.728285601 | 3 | -1.728285601 | Proteins of >= 10-fold downregulation in hypertonic EVs |
| P43274 | 1114792380 | 44764751.01 | 9.047193991 | 7.650936173 | -1.396257818 | Proteins of >= 10-fold downregulation in hypertonic EVs |
| P62918 | 6023896 | 253193.8235 | 6.779877465 | 5.403453107 | -1.376424358 | Proteins of >= 10-fold downregulation in hypertonic EVs |
| P08228 | 13285131.5 | 773255.8796 | 7.123365858 | 5.888323231 | -1.235042627 | Proteins of >= 10-fold downregulation in hypertonic EVs |
| A2AH85 | 1302927.875 | 93062.18753 | 6.114920376 | 4.968773257 | -1.146147119 | Proteins of >= 10-fold downregulation in hypertonic EVs |
| Q5SXR6 | 18899493.69 | 1396814.553 | 7.27645017 | 6.145138751 | -1.131311419 | Proteins of >= 10-fold downregulation in hypertonic EVs |
| P17095 | 2300774.5 | 175500.9731 | 6.361874055 | 5.244279529 | -1.117594526 | Proteins of >= 10-fold downregulation in hypertonic EVs |
| Q9CQF9 | 3199351.031 | 277069.1835 | 6.505061893 | 5.442588225 | -1.062473668 | Proteins of >= 10-fold downregulation in hypertonic EVs |
| Q9DBJ1 | 7092532.5 | 682207.5736 | 6.850801335 | 5.833916536 | -1.016884799 | Proteins of >= 10-fold downregulation in hypertonic EVs |
| P62908 | 8495979.938 | 858189.9559 | 6.929213478 | 5.933583427 | -0.995630051 | Others |
| P15864 | 299224558 | 31713575.38 | 8.475997234 | 7.501245207 | -0.974752027 | Others |
| Q07113 | 3759154.5 | 433892.5336 | 6.575090175 | 5.637382177 | -0.937707998 | Others |
| P47911 | 35186823 | 4105183.817 | 7.546380056 | 6.613332608 | -0.933047448 | Others |
| P29391 | 3777659.125 | 447826.4997 | 6.577222767 | 5.651109789 | -0.926112978 | Others |
| P97429 | 5779061.438 | 708112.8168 | 6.761857312 | 5.850102455 | -0.911754857 | Others |
| P62960 | 6744595.125 | 892674.203 | 6.828955884 | 5.950692984 | -0.8782629 | Others |
| Q8BJ56 | 2435908.25 | 329704.4846 | 6.386660926 | 5.518124854 | -0.868536072 | Others |
| P37889 | 6896750.125 | 954982.9124 | 6.838644491 | 5.979995601 | -0.85864889 | Others |
| P52480 | 11834940.56 | 1647463.273 | 7.073166081 | 6.216815742 | -0.856350339 | Others |
| Q91VA7 | 2523129.875 | 351390.9286 | 6.401939606 | 5.545790546 | -0.85614906 | Others |
| P20152 | 49406042.75 | 6913512.575 | 7.69378007 | 6.839698757 | -0.854081313 | Others |
| Q542V3 | 2390570.5 | 336614.4776 | 6.378501556 | 5.527132791 | -0.851368765 | Others |
| O88844 | 12876141.25 | 1908677.218 | 7.109785732 | 6.28073249 | -0.829053242 | Others |
| Q8BH64 | 3590403.188 | 532630.9794 | 6.555143221 | 5.726426423 | -0.828716798 | Others |
| P18572 | 5476944.25 | 819120.9243 | 6.73853832 | 5.91334802 | -0.8251903 | Others |
| P47962 | 17952850.06 | 2733926.106 | 7.254133404 | 6.436786772 | -0.817346632 | Others |
| E9Q616 | 6673146.422 | 1029175.831 | 6.824330654 | 6.012489579 | -0.811841075 | Others |
| P13020 | 35055559.88 | 5610368.423 | 7.544756908 | 6.748991382 | -0.795765526 | Others |
| P35700 | 26906943.06 | 4359728.607 | 7.42986436 | 6.639459455 | -0.790404905 | Others |
| Q61937 | 3275121.125 | 556174.7322 | 6.515227366 | 5.745211254 | -0.770016112 | Others |
| Q9WV91 | 2827708.969 | 509879.2462 | 6.451434709 | 5.707467335 | -0.743967374 | Others |
| P36536 | 544354.125 | 101503.3907 | 5.735881518 | 5.00648055 | -0.729400968 | Others |
| P07091 | 16027707 | 3327716.326 | 7.204871395 | 6.522146297 | -0.682725098 | Others |
| Q3TEA8 | 3370081.5 | 782315.0626 | 6.527640404 | 5.893381692 | -0.634258712 | Others |
| Q9D819 | 11031760.5 | 2640208.47 | 7.042644825 | 6.42163822 | -0.621006605 | Others |
| P48678 | 21834297.31 | 5589800.942 | 7.33913922 | 6.747396343 | -0.591742877 | Others |
| P14069 | 8639697 | 2226513.024 | 6.936498512 | 6.34762524 | -0.588873272 | Others |
| Q99PT1 | 5179611 | 1334855.913 | 6.714297145 | 6.12543439 | -0.588862755 | Others |
| Q9JIZ9 | 2350266.438 | 616506.3769 | 6.371117099 | 5.789937573 | -0.581179526 | Others |
| A0A1Y7VKY1 | 5383245.625 | 1418892.076 | 6.731044196 | 6.151949363 | -0.579094833 | Others |
| Q60692 | 1800103.5 | 475372.3443 | 6.255297476 | 5.677033912 | -0.578263564 | Others |
| Q9WTI7 | 9901997.813 | 2732454.977 | 6.995722826 | 6.436553015 | -0.559169811 | Others |
| G3X977 | 1592603.375 | 442290.5558 | 6.202107632 | 5.645707666 | -0.556399966 | Others |
| P10107 | 13391512.67 | 3745716.098 | 7.126829637 | 6.573534857 | -0.55329478 | Others |
| Q62261 | 1339276.375 | 383119.59 | 6.126870208 | 5.583334359 | -0.543535849 | Others |
| Q9CRD2 | 1237235.875 | 354664.0021 | 6.092452504 | 5.54981711 | -0.542635394 | Others |
| P62754 | 43699593.38 | 12546018.61 | 7.640477396 | 7.098505927 | -0.541971469 | Others |
| P07901 | 1099440.063 | 315861.5441 | 6.041171558 | 5.499496754 | -0.541674804 | Others |
| Q91XV3 | 860692.5 | 247782.021 | 5.934848019 | 5.394069791 | -0.540778228 | Others |
| Q91VJ2 | 585672.875 | 169331.5611 | 5.76765511 | 5.228737912 | -0.538917198 | Others |
| Q05793 | 16682053.88 | 4899264.231 | 7.22224952 | 6.690130863 | -0.532118657 | Others |
| P24270 | 1736866.125 | 510379.128 | 6.239766345 | 5.707892906 | -0.531873439 | Others |
| Q61753 | 7532471.5 | 2227751.401 | 6.876937497 | 6.347866725 | -0.529070772 | Others |
| Q99LU0 | 3491938.75 | 1044174.894 | 6.543066617 | 6.018773247 | -0.52429337 | Others |
| Q9WU78 | 5489247.688 | 1658542.264 | 6.739512828 | 6.219726543 | -0.519786285 | Others |
| E9Q3X0 | 73764418.34 | 22758165.59 | 7.867846922 | 7.357137253 | -0.510709669 | Others |
| A0A0N4SW28 | 9360782.125 | 2958754.939 | 6.971312137 | 6.471108996 | -0.500203141 | Others |
| Q64727 | 3523260.391 | 1116248.401 | 6.546944741 | 6.04776085 | -0.499183891 | Others |
| Q9QZQ8 | 173124545.5 | 56881791.62 | 8.238358646 | 7.754973267 | -0.483385379 | Others |
| P47963 | 17611844.75 | 5792460.243 | 7.245804848 | 6.762863062 | -0.482941786 | Others |
| A6ZI44 | 42307153.75 | 14092167.25 | 7.626413809 | 7.148977789 | -0.47743602 | Others |
| O09167 | 1807430 | 605509.5343 | 6.257061486 | 5.782120986 | -0.4749405 | Others |
| A0A1B0GSX0 | 2670294.25 | 894718.3123 | 6.426559121 | 5.951686326 | -0.474872795 | Others |
| P70168 | 1920784.102 | 648584.4657 | 6.283478552 | 5.811966543 | -0.471512009 | Others |
| P62702 | 15769841.55 | 5375288.323 | 7.19782733 | 6.730401764 | -0.467425566 | Others |
| P09528 | 3826429.75 | 1313913.302 | 6.582793744 | 6.118566709 | -0.464227035 | Others |
| P60710 | 124400806.6 | 43557702.41 | 8.094823196 | 7.639064963 | -0.455758233 | Others |
| C0HKE6 | 1742221451 | 616590676.9 | 9.241103357 | 8.789996954 | -0.451106403 | Others |
| P46638 | 833764.5625 | 303344.1343 | 5.921043432 | 5.481935602 | -0.43910783 | Others |
| P21956 | 35572746.31 | 13047909.89 | 7.551117395 | 7.115540949 | -0.435576446 | Others |
| Q6IRU2 | 792972.6875 | 291601.1166 | 5.899258229 | 5.464789183 | -0.434469046 | Others |
| P80318 | 1072019.125 | 395263.9336 | 6.030202533 | 5.596887188 | -0.433315345 | Others |
| E9QAZ2 | 27130779.5 | 10488507.94 | 7.433462272 | 7.020713711 | -0.412748561 | Others |
| P12970 | 17670599.97 | 6931133.327 | 7.247251295 | 6.840804253 | -0.406447042 | Others |
| P07356 | 70697242.08 | 28373734.14 | 7.849402472 | 7.452916495 | -0.396485977 | Others |
| Q60847 | 3636092 | 1460874.207 | 6.560634863 | 6.164612821 | -0.396022042 | Others |
| F8VQJ3 | 9779310.75 | 3954090.955 | 6.990308247 | 6.597046655 | -0.393261592 | Others |
| E9Q390 | 1381494.5 | 563619.7123 | 6.14034916 | 5.750986174 | -0.389362986 | Others |
| Q99M71 | 1405786.25 | 582962.4218 | 6.147919291 | 5.765640561 | -0.38227873 | Others |
| Q9WUM3 | 1552802.25 | 647301.5064 | 6.191116152 | 5.811106618 | -0.380009534 | Others |
| H7BX95 | 5280135.75 | 2216611.314 | 6.722645088 | 6.345689546 | -0.376955542 | Others |
| P35980 | 49744787.94 | 21012043.15 | 7.696747584 | 7.322468284 | -0.3742793 | Others |
| P62259 | 4716898.75 | 1994623.936 | 6.673656554 | 6.299861026 | -0.373795528 | Others |
| P26645 | 1490111.813 | 634592.1833 | 6.173218858 | 5.802494718 | -0.37072414 | Others |
| P17182 | 16255074.31 | 7136202.957 | 7.210988959 | 6.853467193 | -0.357521766 | Others |
| P62911 | 23264386.38 | 10421043.43 | 7.366691602 | 7.017911206 | -0.348780396 | Others |
| Q6P4T2 | 379790.9063 | 173776.3842 | 5.579544562 | 5.239990757 | -0.339553805 | Others |
| Q8R5L1 | 2822536 | 1316401.511 | 6.45063949 | 6.119388372 | -0.331251118 | Others |
| P62071 | 1050351.906 | 492289.0859 | 6.021334828 | 5.692220208 | -0.32911462 | Others |
| P09405 | 3963304.234 | 1857679.16 | 6.598057411 | 6.268970709 | -0.329086702 | Others |
| P02535 | 61121240.67 | 29195568.77 | 7.786192161 | 7.46531694 | -0.320875221 | Others |
| P14152 | 5360241 | 2635332.831 | 6.729184316 | 6.420835473 | -0.308348843 | Others |
| E9PZF0 | 4495011.25 | 2223039.844 | 6.652730783 | 6.346947247 | -0.305783536 | Others |
| Q6ZWZ7 | 2325135.875 | 1153701.005 | 6.366448337 | 6.062093271 | -0.304355066 | Others |
| A0A0A0MQF6 | 18953976.69 | 9606018.491 | 7.277700342 | 6.982543418 | -0.295156924 | Others |
| P14869 | 1134178.25 | 597234.8194 | 6.054681315 | 5.77614512 | -0.278536195 | Others |
| Q9QZD8 | 673873.25 | 357256.6828 | 5.828578217 | 5.552980361 | -0.275597856 | Others |
| A0A1W2P768 | 6661653349 | 3565125133 | 9.82358203 | 9.552074778 | -0.271507252 | Others |
| Q04750 | 4196348.5 | 2270905.828 | 6.622871548 | 6.356199125 | -0.266672423 | Others |
| Q4VWZ5 | 1791048.875 | 969662.1125 | 6.253107437 | 5.986620427 | -0.26648701 | Others |
| Q9D8E6 | 13153843.69 | 7279653.898 | 7.119052677 | 6.862110732 | -0.256941945 | Others |
| D3Z1V4 | 979697.75 | 545269.7069 | 5.991092111 | 5.736611371 | -0.25448074 | Others |
| P27659 | 39275392.94 | 22017433.62 | 7.594120539 | 7.342766696 | -0.251353843 | Others |
| O54833 | 937934.375 | 532903.6928 | 5.972172453 | 5.72664873 | -0.245523723 | Others |
| O88322 | 7785993.313 | 4534318.563 | 6.891314026 | 6.656512029 | -0.234801997 | Others |
| P09411 | 10883873.88 | 6481545.25 | 7.036783501 | 6.811678557 | -0.225104944 | Others |
| P11087 | 108279405.5 | 64536263.51 | 8.034545863 | 7.809803817 | -0.224742046 | Others |
| O54962 | 7053311.125 | 4210153.895 | 6.848393041 | 6.624297971 | -0.22409507 | Others |
| P09055 | 3389175.219 | 2086752.613 | 6.530094022 | 6.319470966 | -0.210623056 | Others |
| P14115 | 9001083 | 5544490.052 | 6.954294766 | 6.743861609 | -0.210433157 | Others |
| E9PWQ3 | 9849446.703 | 6081396.415 | 6.993411834 | 6.784003314 | -0.20940852 | Others |
| P10493 | 17347174.69 | 10974269.36 | 7.239228752 | 7.040375616 | -0.198853136 | Others |
| P11499 | 10357488.09 | 6984870.37 | 7.015254443 | 6.844158351 | -0.171096092 | Others |
| P07724 | 68811832 | 46498527.58 | 7.83766312 | 7.667439201 | -0.170223919 | Others |
| Q3UKW2 | 12127404.69 | 8199376.695 | 7.08376787 | 6.913780839 | -0.169987031 | Others |
| Q6GSS7 | 13608503.5 | 9252629.94 | 7.133810369 | 6.966265193 | -0.167545176 | Others |
| Q99JR5 | 323139.0938 | 221774.2642 | 5.509389503 | 5.345911147 | -0.163478356 | Others |
| P14824 | 49557104.28 | 34497284.64 | 7.695105922 | 7.537784912 | -0.15732101 | Others |
| P62962 | 1112107.25 | 779507.065 | 6.046146672 | 5.891820056 | -0.154326616 | Others |
| O35887 | 393383.8125 | 276643.3417 | 5.594816485 | 5.441920222 | -0.152896263 | Others |
| Q61033 | 57171764 | 40333877.29 | 7.757181592 | 7.605669973 | -0.151511619 | Others |
| P61089 | 625863.25 | 443343.0735 | 5.796479451 | 5.646739928 | -0.149739523 | Others |
| Q03350 | 458743.5938 | 325070.8393 | 5.661570013 | 5.511978013 | -0.149592 | Others |
| P61982 | 1516330.625 | 1079635.073 | 6.180793906 | 6.033276985 | -0.147516921 | Others |
| Q01853 | 28259323.06 | 20565364.88 | 7.451161754 | 7.313136419 | -0.138025335 | Others |
| Q8CGP1 | 7287758654 | 5332926753 | 9.862593982 | 9.726965619 | -0.135628363 | Others |
| P62855 | 38462829.13 | 28642585.94 | 7.585041226 | 7.457012225 | -0.128029001 | Others |
| Q01149 | 27550703.7 | 20648630.33 | 7.440132696 | 7.314891249 | -0.125241447 | Others |
| P05213 | 14620861.88 | 11236716.29 | 7.164972974 | 7.050639416 | -0.114333558 | Others |
| Q9CYR0 | 2334178.75 | 1797263.249 | 6.368134111 | 6.254611694 | -0.113522417 | Others |
| Q9R045 | 1216550.375 | 941538.7522 | 6.085130097 | 5.9738382 | -0.111291897 | Others |
| P53986 | 973747.5 | 764116.6026 | 5.988446356 | 5.883159636 | -0.10528672 | Others |
| P06684 | 1587812.375 | 1260805.042 | 6.200799182 | 6.100647937 | -0.100151245 | Others |
| Q8K354 | 2696661.313 | 2168030.26 | 6.430826405 | 6.33606534 | -0.094761065 | Others |
| P50516 | 1851680.25 | 1509266.664 | 6.267565994 | 6.17876598 | -0.088800014 | Others |
| P97807 | 1625248.625 | 1335750.04 | 6.210919807 | 6.125725196 | -0.085194611 | Others |
| P01942 | 26038031.75 | 21417720.64 | 7.415608152 | 7.33077325 | -0.084834902 | Others |
| P41105 | 14464067.69 | 11917942.07 | 7.160290446 | 7.07620127 | -0.084089176 | Others |
| P48036 | 78974881.25 | 65851105.77 | 7.897488982 | 7.818563072 | -0.07892591 | Others |
| P31428 | 1717826.125 | 1460005.238 | 6.234979203 | 6.164354414 | -0.070624789 | Others |
| Q9DBL9 | 1636659.75 | 1416299.909 | 6.213958402 | 6.151155227 | -0.062803175 | Others |
| P17751 | 1838793.563 | 1592733.804 | 6.264532975 | 6.202143198 | -0.062389777 | Others |
| P40142 | 8055444.563 | 6990952.297 | 6.906089513 | 6.844536339 | -0.061553174 | Others |
| Q9WUM5 | 1204380.125 | 1048039.516 | 6.08076358 | 6.020377658 | -0.060385922 | Others |
| P08752 | 3600981.313 | 3267920.823 | 6.556420868 | 6.514271526 | -0.042149342 | Others |
| Q91VR2 | 1934046 | 1762665.964 | 6.286466799 | 6.246170019 | -0.04029678 | Others |
| P10630 | 2582948.75 | 2385447.143 | 6.412115789 | 6.377569798 | -0.034545991 | Others |
| E9PZ16 | 221177404.8 | 205129086.8 | 8.344740758 | 8.312027247 | -0.032713511 | Others |
| P62852 | 3823463.25 | 3584468.365 | 6.58245692 | 6.554424752 | -0.028032168 | Others |
| P53026 | 2036621.875 | 1926365.25 | 6.308910404 | 6.284738635 | -0.024171769 | Others |
| P27773 | 5885440.469 | 5575461.243 | 6.769778971 | 6.746280801 | -0.02349817 | Others |
| Q3UXS0 | 12375435.03 | 11858827.97 | 7.092560474 | 7.074041769 | -0.018518705 | Others |
| Q80YQ1 | 5124060.125 | 4962920.42 | 6.709614217 | 6.695737311 | -0.013876906 | Others |
| E9QN70 | 1254864.219 | 1217670.018 | 6.098596736 | 6.085529613 | -0.013067123 | Others |
| E9QPR6 | 587413030.5 | 582395886.9 | 8.768943576 | 8.765218299 | -0.003725277 | Others |
| A8DUK4 | 19511826 | 19594483.99 | 7.290297914 | 7.292133831 | 0.001835917 | Others |
| Q02053 | 4163314 | 4209250.27 | 6.619439167 | 6.624204748 | 0.004765581 | Others |
| Q99LP6 | 1477563.5 | 1513980.574 | 6.169546154 | 6.180120303 | 0.010574149 | Others |
| Q9CPQ1 | 1779484.438 | 1826372.725 | 6.250294194 | 6.261589413 | 0.011295219 | Others |
| P13707 | 32406050 | 33520474.79 | 7.510626098 | 7.525310161 | 0.014684063 | Others |
| P62806 | 5491896911 | 5705177094 | 9.739722376 | 9.75626913 | 0.016546754 | Others |
| Q04857 | 4385774.688 | 4577833.092 | 6.642046317 | 6.660659954 | 0.018613637 | Others |
| Q9CZ13 | 1740086 | 1816398.536 | 6.240570713 | 6.259211143 | 0.01864043 | Others |
| A0A087WR50 | 488083990.6 | 510560927.4 | 8.688494563 | 8.708047576 | 0.019553013 | Others |
| Q8R1B4 | 1033900.719 | 1091875.769 | 6.014478837 | 6.038173228 | 0.023694391 | Others |
| Q08857 | 15711117 | 16603663.03 | 7.196207063 | 7.220203911 | 0.023996848 | Others |
| Q6ZWN5 | 7536154.969 | 7992498.21 | 6.87714982 | 6.902682548 | 0.025532728 | Others |
| Q9WVB4 | 2455584.891 | 2619718.259 | 6.390154953 | 6.418254587 | 0.028099634 | Others |
| P50247 | 554685.5625 | 592953.1258 | 5.744046862 | 5.773020363 | 0.028973501 | Others |
| P11438 | 2162011.5 | 2337205.205 | 6.334858 | 6.368696845 | 0.033838845 | Others |
| P10852 | 3940887.188 | 4286148.587 | 6.595594003 | 6.632067223 | 0.03647322 | Others |
| P35505 | 1924905.5 | 2125612.51 | 6.284409413 | 6.327484097 | 0.043074684 | Others |
| P37040 | 2243634.688 | 2534066.759 | 6.350952146 | 6.403818052 | 0.052865906 | Others |
| P63017 | 22897936.19 | 25897829.42 | 7.359796341 | 7.413263366 | 0.053467025 | Others |
| P70372 | 480488.4375 | 544458.3697 | 5.681682941 | 5.735964678 | 0.054281737 | Others |
| P62264 | 447942.3125 | 509025.5944 | 5.651222088 | 5.70673962 | 0.055517532 | Others |
| P17742 | 8649651.375 | 9899792.266 | 6.936998604 | 6.995626082 | 0.058627478 | Others |
| P10854 | 63481566.25 | 74769692.84 | 7.802647633 | 7.873725597 | 0.071077964 | Others |
| P62242 | 76252490.13 | 91777750.73 | 7.882254031 | 7.96273741 | 0.080483379 | Others |
| Q99JY0 | 3827153.063 | 4636719.322 | 6.582875832 | 6.666210807 | 0.083334975 | Others |
| P10639 | 1694934.75 | 2059286.777 | 6.229152984 | 6.313716831 | 0.084563847 | Others |
| Q922U2 | 4090925.25 | 4983263 | 6.611821544 | 6.697513808 | 0.085692264 | Others |
| Q68FL4 | 143956.6719 | 181629.8033 | 5.158231798 | 5.259187113 | 0.100955315 | Others |
| Q921K2 | 5349036 | 6813156.632 | 6.728275521 | 6.833348373 | 0.105072852 | Others |
| Q8BKZ9 | 4823489.75 | 6226484.94 | 6.68336136 | 6.794242942 | 0.110881582 | Others |
| G3UYX7 | 1143560.281 | 1476789.249 | 6.058259063 | 6.169318522 | 0.111059459 | Others |
| O09159 | 36534376.13 | 47565514.22 | 7.562701696 | 7.677292196 | 0.1145905 | Others |
| P14206 | 27182477.5 | 35642258.99 | 7.434289037 | 7.551965222 | 0.117676185 | Others |
| Q8CGN5 | 4893312.875 | 6426220.638 | 6.689602985 | 6.807955633 | 0.118352648 | Others |
| Q62376 | 2046164.563 | 2697366.516 | 6.310940559 | 6.430939962 | 0.119999403 | Others |
| P27661 | 4454742078 | 5905283025 | 9.648822564 | 9.771240717 | 0.122418153 | Others |
| P51660 | 5996172.438 | 8004436.799 | 6.777874114 | 6.90333078 | 0.125456666 | Others |
| P08121 | 261199.4219 | 350651.9413 | 5.416972211 | 5.544876248 | 0.127904037 | Others |
| A0A1D5RM85 | 2604535.063 | 3505720.697 | 6.415730208 | 6.544777313 | 0.129047105 | Others |
| A0A0R4J117 | 1035891.063 | 1421382.201 | 6.015314086 | 6.152710873 | 0.137396787 | Others |
| O88783 | 2220162.125 | 3197508.241 | 6.34638469 | 6.504811672 | 0.158426982 | Others |
| P62267 | 1610673 | 2349381.681 | 6.207007379 | 6.370953578 | 0.163946199 | Others |
| P0C0S6 | 27742137.36 | 41185499.02 | 7.443139918 | 7.614744332 | 0.171604414 | Others |
| P51150 | 2366953.625 | 3586853.73 | 6.374189749 | 6.554713667 | 0.180523918 | Others |
| Q60994 | 9369113.5 | 14225491.09 | 6.9716985 | 7.153067268 | 0.181368768 | Others |
| P10126 | 4798228.281 | 7300222.384 | 6.681080906 | 6.86333609 | 0.182255184 | Others |
| G5E866 | 1002057.375 | 1526427.838 | 6.000892589 | 6.183676278 | 0.182783689 | Others |
| Q9Z331 | 14499173 | 22610319.45 | 7.161343232 | 7.354306698 | 0.192963466 | Others |
| P84099 | 5320040.125 | 8330835.223 | 6.725914908 | 6.920688545 | 0.194773637 | Others |
| Q61335 | 3076600.5 | 4943881.721 | 6.488071106 | 6.694068072 | 0.205996966 | Others |
| Q99K41 | 24671470.67 | 40510652.7 | 7.392195039 | 7.607569241 | 0.215374202 | Others |
| G3X8R0 | 1789364.875 | 2952242.165 | 6.252698908 | 6.470151979 | 0.217453071 | Others |
| Q8VEK3 | 18777702.72 | 30994479.88 | 7.273642459 | 7.491284353 | 0.217641894 | Others |
| P19096 | 4187557.484 | 6932036.46 | 6.621960782 | 6.840860838 | 0.218900056 | Others |
| Q8VDD5 | 4130289.672 | 6882535.864 | 6.615980511 | 6.837748483 | 0.221767972 | Others |
| P04104 | 29922297.89 | 50094901.89 | 7.475994942 | 7.69979353 | 0.223798588 | Others |
| P04117 | 50501923.81 | 84801952.46 | 7.703307922 | 7.928405851 | 0.225097929 | Others |
| P68372 | 4225335.297 | 7329669.713 | 6.625861178 | 6.865084405 | 0.239223227 | Others |
| P35564 | 2724839.516 | 4736305.472 | 6.435340929 | 6.675439705 | 0.240098776 | Others |
| P19221 | 2086478.938 | 3662051.846 | 6.319414005 | 6.563724489 | 0.244310484 | Others |
| P86048 | 2195747.805 | 3917212.53 | 6.341582457 | 6.592977135 | 0.251394678 | Others |
| Q3TTY5 | 5297984.828 | 9689600.842 | 6.72411071 | 6.986305887 | 0.262195177 | Others |
| E9QNP0 | 16134695.75 | 29589284.55 | 7.20776078 | 7.471134464 | 0.263373684 | Others |
| A0A0R4J1G5 | 326909.5313 | 628861.4807 | 5.514427583 | 5.798554994 | 0.284127411 | Others |
| P61750 | 301124.1875 | 587705.8541 | 5.478745641 | 5.769160017 | 0.290414376 | Others |
| Q9D1R9 | 1136328.75 | 2226672.015 | 6.055503995 | 6.347656251 | 0.292152256 | Others |
| P14148 | 16364220.88 | 32203388.4 | 7.213895333 | 7.50790157 | 0.294006237 | Others |
| Q03265 | 40868611.39 | 81240961.92 | 7.611389882 | 7.909775057 | 0.298385175 | Others |
| P19783 | 814995.75 | 1643866.914 | 5.911155344 | 6.215866655 | 0.304711311 | Others |
| Q60930 | 6351413.5 | 12887191.48 | 6.802870388 | 7.110158281 | 0.307287893 | Others |
| P38647 | 17584744.84 | 36305653.7 | 7.245136071 | 7.559974261 | 0.31483819 | Others |
| Q64191 | 1232268.25 | 2602246.122 | 6.090705259 | 6.41534837 | 0.324643111 | Others |
| P08113 | 3672043.625 | 7858372.641 | 6.564907832 | 6.895332619 | 0.330424787 | Others |
| P97857 | 94865.51563 | 203882.2323 | 4.977108372 | 5.30937938 | 0.332271008 | Others |
| Q792Y8 | 3382459.281 | 7414427.343 | 6.529232577 | 6.870077614 | 0.340845037 | Others |
| B1AQ77 | 12236655.59 | 27323844.46 | 7.087662737 | 7.436541804 | 0.348879067 | Others |
| Q9CR57 | 8817745.5 | 19743499.97 | 6.94535756 | 7.295424143 | 0.350066583 | Others |
| Q9D6Z1 | 967394.9063 | 2177499.069 | 5.985603796 | 6.337957978 | 0.352354182 | Others |
| O35129 | 1503624.906 | 3400485.008 | 6.177139511 | 6.531540864 | 0.354401353 | Others |
| P20029 | 29990546.13 | 69797511.63 | 7.476984374 | 7.84383994 | 0.366855566 | Others |
| E9QPX1 | 837590.125 | 1989753.517 | 5.923031548 | 6.298799281 | 0.375767733 | Others |
| P17047 | 2112896.781 | 5341837.084 | 6.324878281 | 6.727690639 | 0.402812358 | Others |
| D3YUE2 | 1016930.469 | 2595238.578 | 6.00729126 | 6.414177288 | 0.406886028 | Others |
| Q8BP67 | 1288888.281 | 3456415.289 | 6.110215275 | 6.538625918 | 0.428410643 | Others |
| Q3TML0 | 1694647.625 | 4549362.23 | 6.229079407 | 6.657950518 | 0.428871111 | Others |
| D3Z041 | 47014617.19 | 127487291 | 7.672232904 | 8.105466893 | 0.433233989 | Others |
| P62082 | 184450096 | 503598782.5 | 8.265878886 | 8.702084671 | 0.436205785 | Others |
| E9Q4M2 | 125972.7734 | 346312.9154 | 5.100276691 | 5.539468688 | 0.439191997 | Others |
| H3BJQ7 | 632882.9375 | 1746591.108 | 5.801323387 | 6.242191245 | 0.440867858 | Others |
| Q02788 | 1359676.5 | 3896130.513 | 6.133435591 | 6.590633496 | 0.457197905 | Others |
| Q80YX1 | 854146.6875 | 2472125.575 | 5.931532461 | 6.393070528 | 0.461538067 | Others |
| P97351 | 8784158.031 | 25659343.79 | 6.94370014 | 7.409245546 | 0.465545406 | Others |
| Q3THE2 | 98695.16406 | 290125.1316 | 4.994295873 | 5.46258535 | 0.468289477 | Others |
| P01831 | 513337.25 | 1523980.777 | 5.71040278 | 6.182979489 | 0.472576709 | Others |
| Q99JR1 | 1733137.938 | 5146380.256 | 6.238833129 | 6.711501872 | 0.472668743 | Others |
| P56395 | 3457268 | 10283474.4 | 6.538733046 | 7.012139871 | 0.473406825 | Others |
| P97927 | 4003099.469 | 12093580.16 | 6.602396382 | 7.082554888 | 0.480158506 | Others |
| P11152 | 50164889.06 | 154374640.1 | 7.700399856 | 8.188575958 | 0.488176102 | Others |
| E9Q600 | 104327.6484 | 330721.5761 | 5.018399418 | 5.519462529 | 0.501063111 | Others |
| Q99KI0 | 7838939.344 | 25515718.99 | 6.894257304 | 7.406807811 | 0.512550507 | Others |
| O35988 | 3885113.75 | 12988590.87 | 6.589403739 | 7.113562037 | 0.524158298 | Others |
| E9QPD7 | 24850914.32 | 86184483.17 | 7.395342372 | 7.935429082 | 0.54008671 | Others |
| Q8BX10 | 667970.375 | 2356112.874 | 5.824757202 | 6.372196092 | 0.54743889 | Others |
| Q9DCZ4 | 248981.0625 | 891742.1686 | 5.396166316 | 5.950239304 | 0.554072988 | Others |
| Q9Z0X1 | 209627.5469 | 760625.5808 | 5.321448352 | 5.881170927 | 0.559722575 | Others |
| Q8BMF4 | 24548893.75 | 94441933.07 | 7.390031926 | 7.975164868 | 0.585132942 | Others |
| Q3V117 | 847785.875 | 3328393.531 | 5.928286176 | 6.522234669 | 0.593948493 | Others |
| Q9D880 | 283271.8438 | 1112212.979 | 5.452203409 | 6.046187959 | 0.59398455 | Others |
| Q07417 | 1202547.875 | 5083953.802 | 6.080102375 | 6.706201595 | 0.62609922 | Others |
| P56382 | 3083781.75 | 13055367.39 | 6.489083634 | 7.115789098 | 0.626705464 | Others |
| Q00623 | 2254890.75 | 9571884.763 | 6.353125505 | 6.980997461 | 0.627871956 | Others |
| Q64433 | 1287120.625 | 5742912.755 | 6.10961925 | 6.759132219 | 0.649512969 | Others |
| P05202 | 2209000.875 | 9883930.352 | 6.344195888 | 6.994929676 | 0.650733788 | Others |
| Q9D2G2 | 2361485.5 | 10685385.58 | 6.373185283 | 7.028790198 | 0.655604915 | Others |
| E9Q0F0 | 9835902 | 45250027.24 | 6.992814193 | 7.655618845 | 0.662804652 | Others |
| O55143 | 3969393.5 | 18600915.43 | 6.598724154 | 7.269534318 | 0.670810164 | Others |
| Q02105 | 901486.875 | 4400733.886 | 5.954959408 | 6.643525107 | 0.688565699 | Others |
| P12787 | 2651056 | 13192761.17 | 6.423418902 | 7.1203357 | 0.696916798 | Others |
| P26041 | 625248651 | 3248979534 | 8.796052763 | 9.511746976 | 0.715694213 | Others |
| P70452 | 859336.625 | 4492900.898 | 5.934163322 | 6.652526839 | 0.718363517 | Others |
| O70503 | 3106061.063 | 16928241.88 | 6.492209989 | 7.228611856 | 0.736401867 | Others |
| Q8VCT4 | 12073022.38 | 66479221.58 | 7.081816006 | 7.822685926 | 0.74086992 | Others |
| Q9DCW4 | 5180304.563 | 28901374.21 | 6.714355294 | 7.460918493 | 0.746563199 | Others |
| A0A1L1STE6 | 3170993.469 | 17972746.13 | 6.501195348 | 7.25461444 | 0.753419092 | Others |
| Q91YQ5 | 362402.4375 | 2057765.033 | 5.55919111 | 6.313395783 | 0.754204673 | Others |
| Q60932 | 1275585.281 | 7588749.272 | 6.105709499 | 6.880170204 | 0.774460705 | Others |
| Q9WTM5 | 242371.5781 | 1450611.132 | 5.384481691 | 6.161551006 | 0.777069315 | Others |
| O35855 | 884476.5625 | 5578342.96 | 5.946686329 | 6.746505211 | 0.799818882 | Others |
| Q60597 | 1829495.5 | 11856433.83 | 6.262331346 | 7.073954082 | 0.811622736 | Others |
| P08249 | 14381576.25 | 93917000.88 | 7.157806488 | 7.972744215 | 0.814937727 | Others |
| P41731 | 825872.8125 | 5451643.952 | 5.916913169 | 6.736527484 | 0.819614315 | Others |
| Q5SWU9 | 548163.75 | 3942515.228 | 5.738910312 | 6.595773379 | 0.856863067 | Others |
| Q920S1 | 38694145.5 | 281114044.9 | 7.58764526 | 8.448882544 | 0.861237284 | Others |
| P62897 | 415482.6875 | 3204413.099 | 5.618552932 | 6.505748498 | 0.887195566 | Others |
| Q9CZU6 | 3746296.656 | 30931456.02 | 6.573602165 | 7.490400364 | 0.916798199 | Others |
| P32020 | 244402.4844 | 2041366.079 | 5.388105616 | 6.309920894 | 0.921815278 | Others |
| P03930 | 1747142.625 | 14806298.29 | 6.242328359 | 7.170446494 | 0.928118135 | Others |
| P45952 | 6847287.063 | 60728024.1 | 6.835518535 | 7.783389151 | 0.947870616 | Others |
| Q9EQ20 | 530732.75 | 5028058.434 | 5.724875888 | 6.701400316 | 0.976524428 | Others |
| Q99P88 | 1435889.125 | 13687038.23 | 6.157120906 | 7.13630948 | 0.979188574 | Others |
| Q8JZR0 | 762465.625 | 7511957.918 | 5.882220269 | 6.875753146 | 0.993532877 | Others |
| P56480 | 7208335.516 | 73705005.51 | 6.857834993 | 7.867496983 | 1.00966199 | Proteins of >= 10-fold upregulation in hypertonic EVs |
| Q9QZF2 | 1135269.875 | 11792810.2 | 6.055099114 | 7.071617309 | 1.016518195 | Proteins of >= 10-fold upregulation in hypertonic EVs |
| Q99LC5 | 600654.4375 | 6726948.923 | 5.77862469 | 6.82781813 | 1.04919344 | Proteins of >= 10-fold upregulation in hypertonic EVs |
| P51881 | 5039710.047 | 57426069.6 | 6.702405551 | 7.759109093 | 1.056703542 | Proteins of >= 10-fold upregulation in hypertonic EVs |
| P67778 | 3918989.188 | 45195236.25 | 6.593174065 | 7.655092661 | 1.061918596 | Proteins of >= 10-fold upregulation in hypertonic EVs |
| P51655 | 598249.9375 | 7333571.388 | 5.776882662 | 6.865315524 | 1.088432862 | Proteins of >= 10-fold upregulation in hypertonic EVs |
| Q9CRB9 | 76904.42969 | 945044.1498 | 4.885951356 | 5.975452098 | 1.089500742 | Proteins of >= 10-fold upregulation in hypertonic EVs |
| P50544 | 1002069.125 | 12817573.33 | 6.000897681 | 7.107805811 | 1.10690813 | Proteins of >= 10-fold upregulation in hypertonic EVs |
| P35486 | 288440.8906 | 3775526.096 | 5.460056828 | 6.576977476 | 1.116920648 | Proteins of >= 10-fold upregulation in hypertonic EVs |
| Q9CQ65 | 189012.7656 | 2535672.063 | 5.276491137 | 6.404093086 | 1.127601949 | Proteins of >= 10-fold upregulation in hypertonic EVs |
| Q8BWT1 | 626527.3438 | 8434394.799 | 5.79694003 | 6.926053926 | 1.129113896 | Proteins of >= 10-fold upregulation in hypertonic EVs |
| Q3UV17 | 8987431 | 132948115 | 6.953635569 | 8.123682184 | 1.170046615 | Proteins of >= 10-fold upregulation in hypertonic EVs |
| Q99LD8 | 319225.9375 | 4924614.934 | 5.504098171 | 6.692372278 | 1.188274107 | Proteins of >= 10-fold upregulation in hypertonic EVs |
| Q9CQQ7 | 510010.0313 | 8181823.351 | 5.707578718 | 6.912850099 | 1.205271381 | Proteins of >= 10-fold upregulation in hypertonic EVs |
| Q9CQ69 | 169198.8125 | 2825450.227 | 5.228397311 | 6.451087661 | 1.22269035 | Proteins of >= 10-fold upregulation in hypertonic EVs |
| Q62425 | 439492.3125 | 8294000.182 | 5.642951283 | 6.918764041 | 1.275812758 | Proteins of >= 10-fold upregulation in hypertonic EVs |
| Q9D051 | 646371.4688 | 12311174 | 5.810482178 | 7.090299469 | 1.279817291 | Proteins of >= 10-fold upregulation in hypertonic EVs |
| P26443 | 247766.9688 | 5596381.66 | 5.394043408 | 6.747907325 | 1.353863917 | Proteins of >= 10-fold upregulation in hypertonic EVs |
| A0A0U1RP81 | 166531.8438 | 3790659.248 | 5.22149729 | 6.578714746 | 1.357217456 | Proteins of >= 10-fold upregulation in hypertonic EVs |
| Q9DB77 | 124904.2969 | 2881833.005 | 5.096577379 | 6.459668811 | 1.363091432 | Proteins of >= 10-fold upregulation in hypertonic EVs |
| Q8BTM8 | 4103329.875 | 142454473.1 | 6.613136432 | 8.153676091 | 1.540539659 | Proteins of >= 10-fold upregulation in hypertonic EVs |
| Q8BMS1 | 1219408.391 | 56277694.34 | 6.086149179 | 7.750336296 | 1.664187117 | Proteins of >= 10-fold upregulation in hypertonic EVs |
| P63038 | 1213965.563 | 68073389.12 | 6.084206367 | 7.832977373 | 1.748771006 | Proteins of >= 10-fold upregulation in hypertonic EVs |
| P48758 | 32054728 | 2366851031 | 7.505892096 | 9.374170924 | 1.868278828 | Proteins of >= 10-fold upregulation in hypertonic EVs |
| Q8R2Q8 | 1000 | 84566.01597 | 3 | 4.927195871 | 1.927195871 | Proteins of >= 10-fold upregulation in hypertonic EVs |
| P43406 | 1000 | 123282.2509 | 3 | 5.090900555 | 2.090900555 | Proteins of >= 10-fold upregulation in hypertonic EVs |
| Q9D7N9 | 163345.25 | 37968426.45 | 5.21310651 | 7.579422599 | 2.366316089 | Proteins of >= 10-fold upregulation in hypertonic EVs |
| P18654 | 1000 | 650047.2514 | 3 | 5.812944926 | 2.812944926 | Proteins of >= 10-fold upregulation in hypertonic EVs |
| A0A0A0MQM7 | 1000 | 687391.4375 | 3 | 5.837204118 | 2.837204118 | Proteins of >= 10-fold upregulation in hypertonic EVs |
| Q61292 | 1000 | 689333.0333 | 3 | 5.838429091 | 2.838429091 | Proteins of >= 10-fold upregulation in hypertonic EVs |
| Q9CQ54 | 1000 | 1150695.938 | 3 | 6.06096058 | 3.06096058 | Proteins of >= 10-fold upregulation in hypertonic EVs |
| P62281 | 1000 | 1187624.765 | 3 | 6.074679245 | 3.074679245 | Proteins of >= 10-fold upregulation in hypertonic EVs |
| Q61781 | 1000 | 1403725.916 | 3 | 6.147282318 | 3.147282318 | Proteins of >= 10-fold upregulation in hypertonic EVs |
| O35216 | 1000 | 1419112.356 | 3 | 6.152016781 | 3.152016781 | Proteins of >= 10-fold upregulation in hypertonic EVs |
| P62821 | 1000 | 1514953.169 | 3 | 6.180399208 | 3.180399208 | Proteins of >= 10-fold upregulation in hypertonic EVs |
| Q6IFX2 | 1000 | 1727818.127 | 3 | 6.237498026 | 3.237498026 | Proteins of >= 10-fold upregulation in hypertonic EVs |
| Q9Z2I0 | 1000 | 1814756.95 | 3 | 6.258818468 | 3.258818468 | Proteins of >= 10-fold upregulation in hypertonic EVs |
| Q9Z2I9 | 1000 | 1942676.366 | 3 | 6.288400457 | 3.288400457 | Proteins of >= 10-fold upregulation in hypertonic EVs |
| E9QKR0 | 1000 | 2147009.084 | 3 | 6.331833882 | 3.331833882 | Proteins of >= 10-fold upregulation in hypertonic EVs |
| D6REV1 | 1000 | 2196520.204 | 3 | 6.341735202 | 3.341735202 | Proteins of >= 10-fold upregulation in hypertonic EVs |
| Q9DC69 | 1000 | 2837200.091 | 3 | 6.452889965 | 3.452889965 | Proteins of >= 10-fold upregulation in hypertonic EVs |
| Q9DB20 | 1000 | 2892936.297 | 3 | 6.461338871 | 3.461338871 | Proteins of >= 10-fold upregulation in hypertonic EVs |
| Q61425 | 1000 | 4008197.813 | 3 | 6.602949147 | 3.602949147 | Proteins of >= 10-fold upregulation in hypertonic EVs |
| P08730 | 1000 | 4099259.767 | 3 | 6.61270544 | 3.61270544 | Proteins of >= 10-fold upregulation in hypertonic EVs |
| Q9CQX2 | 1000 | 5044199.436 | 3 | 6.702792249 | 3.702792249 | Proteins of >= 10-fold upregulation in hypertonic EVs |
| Q9CQ62 | 1000 | 5496326.497 | 3 | 6.740072523 | 3.740072523 | Proteins of >= 10-fold upregulation in hypertonic EVs |
| Q9Z1Q5 | 1000 | 41441874.89 | 3 | 7.617439395 | 4.617439395 | Proteins of >= 10-fold upregulation in hypertonic EVs |

**Supplementary Table 3.**

The fold change between control and hypertonic group (XLSX).
